# Impact of ASL Exposure on Spoken Phonemic Discrimination in Adult CI Users: A Functional Near-Infrared Spectroscopy Study

**DOI:** 10.1162/nol_a_00143

**Published:** 2024-06-14

**Authors:** Shakhlo Nematova, Benjamin Zinszer, Thierry Morlet, Giovanna Morini, Laura-Ann Petitto, Kaja K. Jasińska

**Affiliations:** Department of Linguistics and Cognitive Science, University of Delaware, Newark, DE, USA; Department of Psychology, Swarthmore College, Swarthmore, PA, USA; Nemours Children’s Hospital, Delaware, Wilmington, DE, USA; Department of Communication Sciences and Disorders, University of Delaware, Newark, DE, USA; Brain and Language Center for Neuroimaging, Gallaudet University, Washington, DC, USA; Department of Applied Psychology and Human Development, University of Toronto, Toronto, Ontario, Canada

**Keywords:** American Sign Language, cochlear implant, fNIRS, language deprivation, signed language, spoken language

## Abstract

We examined the impact of exposure to a signed language (American Sign Language, or ASL) at different ages on the neural systems that support spoken language phonemic discrimination in deaf individuals with cochlear implants (CIs). Deaf CI users (*N* = 18, age = 18–24 yrs) who were exposed to a signed language at different ages and hearing individuals (*N* = 18, age = 18–21 yrs) completed a phonemic discrimination task in a spoken native (English) and non-native (Hindi) language while undergoing functional near-infrared spectroscopy neuroimaging. Behaviorally, deaf CI users who received a CI early versus later in life showed better English phonemic discrimination, albeit phonemic discrimination was poor relative to hearing individuals. Importantly, the age of exposure to ASL was not related to phonemic discrimination. Neurally, early-life language exposure, irrespective of modality, was associated with greater neural activation of left-hemisphere language areas critically involved in phonological processing during the phonemic discrimination task in deaf CI users. In particular, early exposure to ASL was associated with increased activation in the left hemisphere’s classic language regions for native versus non-native language phonemic contrasts for deaf CI users who received a CI later in life. For deaf CI users who received a CI early in life, the age of exposure to ASL was not related to neural activation during phonemic discrimination. Together, the findings suggest that early signed language exposure does not negatively impact spoken language processing in deaf CI users, but may instead potentially offset the negative effects of language deprivation that deaf children without any signed language exposure experience prior to implantation. This empirical evidence aligns with and lends support to recent perspectives regarding the impact of ASL exposure in the context of CI usage.

## INTRODUCTION

Earlier studies have presented conflicting perspectives on the influence of exposure to signed language on the acquisition of spoken language skills by deaf and hard-of-hearing (henceforth “deaf”) individuals who are cochlear implant (CI) users. Previous work has reported that deaf children with CIs who were exposed to a signed language (pre- and post-implant) showed poorer spoken language outcomes compared to children with CIs without exposure to a signed language (Geers et al., 2017). On the one hand, researchers have argued that typical spoken language development requires auditory and oral input early in life. This view indicates that auditory–oral language input yields better spoken language outcomes, and signed language input interferes with spoken language development (Geers et al., 2017). On the other hand, other research evidence suggests that early life exposure to a signed language does not negatively impact spoken language development in young deaf children with CIs and may even offset the potential negative effects of language deprivation that some deaf children experience (i.e., children without any exposure to a signed language) prior to implantation (Davidson et al., 2014; Jasińska & Petitto, 2013; Petitto et al., 2016). This view is further supported by research showing that signed and spoken languages are largely neurobiologically equivalent (i.e., signed and spoken language development have equivalent maturational timelines and are processed by the same neural language networks), and early-life exposure to a signed language provides a strong linguistic foundation for learning a spoken language (Emmorey, 2021; Hickok et al., 1996; MacSweeney et al., 2008; Petitto et al., 2016; Petitto et al., 2000). Recent research and evolving perspectives challenge earlier viewpoints and suggest a more balanced and nuanced understanding. There is a need for further investigation to understand the complex interplay between exposure to signed language and its impact on spoken language abilities via CI.

Abundant evidence shows a close relationship between the age of language exposure and later language outcomes (e.g., Birdsong & Molis, 2001; Cheng et al., 2018). It is well known that individuals, both hearing and deaf, who are exposed to a language later in life show poorer outcomes, particularly in the areas of phonology, morphology, and syntax (e.g., Baker et al., 2008; MacSweeney et al., 2008; Mayberry et al., 2002). It is also worth emphasizing that deaf individuals who receive CIs at an earlier age tend to achieve more favorable spoken language outcomes (e.g., Dettman et al., 2016; Houston et al., 2012; Houston & Miyamoto, 2010). While the significance of the early age of CI implantation is firmly established, less is known about how the timing of signed language exposure in the context of CI usage impacts spoken language processing and its underlying neurobiology. Thus, the goal of this study was to examine the effect of exposure to a signed language *at different ages* on the neural systems that support English language phonemic discrimination in deaf individuals with CIs using functional near-infrared spectroscopy (fNIRS). We pursued this goal from a novel vantage point, examining the part of language traditionally associated with auditory and oral input: phonology, specifically phonemic discrimination. This core property of human language, phonology/phonemic discrimination, is centrally important to the word (morphological), sentence (syntactic), and reading processes of a language (Jasińska & Petitto, 2014). In early life, the baby’s acquisition of this level of language organization is essential to its capacity to “find” words in the constantly varying linguistic sound stream around it, differentiate among words (such as *bin* and *pin*), discern meanings (morphological and lexical), and crack the code for the syntactic patterning of their native language. To be sure, the phonological level of organization is universal to all languages, including the world’s natural signed languages, as the capacity to discern and discriminate among phonemic units in one’s native language is central to the human language capacity to produce and, crucially, to comprehend language, and particularly vital to early reading acquisition in all children, hearing and deaf (Jasińska & Petitto, 2014).

Infants initially possess a broad capacity to discriminate speech sounds from both their home (community) language environment (native-language phonetic/phonemic units) and from a language different from the home/community language (non-native language sounds, or phonetic/phonemic units not found in their native language). This ability evolves over the first 6 to 12 months of life, with infants developing increased sensitivity to native language sounds. After approximately ages 12–14 months, children possess peaked sensitivity to the phonetic/phonemic units of their native language, while their earlier sensitivity to non-native speech sounds now dramatically declines (see Werker & Hensch, 2015, for review). Once the attuned sensitivity to native phonological categories is established, they can use these native phonological categories to segment words and recognize familiar words (Werker & Hensch, 2015). Notably, infants who are exposed to more than one language take longer to establish native phonetic categories and may maintain sensitivity to non-native speech sound differences until an older age compared to monolingual infants (Bosch & Sebastián-Gallés, 2003). This period of sensitivity to non-native contrasts may extend up until 3.5 years of age for infants who have experienced hearing loss and received CIs (Kral & Sharma, 2012; Werker & Hensch, 2015). This is attributed both to the gradual stabilization of the brain structures and neural systems that make possible language processing as well as a decline in selected aspects of brain plasticity over the course of life.

Speech perception in hearing individuals, including phonemic discrimination, is supported by a specialized perisylvian neural circuitry comprising a ventral and a dorsal system (Hickok & Poeppel, 2007). The ventral system is involved in mapping sound to distributed conceptual representations supported by regions in the left temporal lobe (Hickok & Poeppel, 2007; Zatorre & Belin, 2001). The dorsal system is involved in mapping sound to articulatory representations supported by left parietal-temporal regions, including the left superior temporal gyrus (LSTG) and, ultimately, frontal regions such as the left inferior frontal gyrus (LIFG) and Broca’s area, which are involved in morphological and syntactic processing and semantic retrieval (Hickok & Poeppel, 2007). Infants show greater activation in the LSTG (Brodmann area [BA] 21/22) compared to other brain areas during phonemic discrimination in their native language (Petitto et al., 2012), and similarly, adults show greater activation in LSTG and Broca’s area (BA 44/45) during phoneme discrimination, phoneme segmentation, and phonological awareness tasks (Burton & Small, 2006; Zatorre et al., 1996).

The above pattern is not limited to spoken languages or hearing individuals; rather, both signed and spoken language processing share a common neural architecture. Similar to spoken language, signed language processing activates LSTG and the left superior temporal sulcus (LSTS) in addition to the LIFG (MacSweeney et al., 2002; Penhune et al., 2003; Petitto et al., 2000). For example, in studies with deaf participants, activation in the region of Wernicke’s area (BA 21/22 LSTG) was associated with signed language perception, whereas activation in the left prefrontal gyrus in Broca’s area was associated with signed language production (Petitto et al., 2000).

Despite the neurobiological equivalence between spoken and signed language, the effect of exposure to a signed language prior to or while using CI requires further research. It is unclear whether the timing of signed language exposure throughout development has any discernible effects on phoneme discrimination skills in adult CI users. Comparing patterns of neural activation underlying phonemic discrimination in earlier- and later-sign-exposed CI users can provide new insights into how signed language impacts spoken language processing in the brain and reveal whether signed language exposure may engage or impede classic left-hemisphere language networks during spoken language processing. Much of the evidence on the impact of exposure to a signed language on spoken language development comes, almost exclusively, from studies of young CI users’ spoken language outcomes. However, the existing behavioral literature remains inconsistent with regard to whether early exposure to a signed language or simultaneous signed and spoken language exposure impedes or facilitates the development of a spoken language during CI use.

### Behavioral Studies on the Effect of Early Exposure to a Signed Language on Deaf CI Users’ Language Development

#### Early exposure to a signed language impedes spoken language outcomes

Exposure to a signed language before implantation has been argued to trigger cross-modal plasticity, which is the brain’s capacity to reorganize a sensory deficient cortical region to process a different sensory modality, and, in the case of deafness, reduce the capacity of the auditory cortex to process speech (Cardin et al., 2020; Giraud & Lee, 2007; Lee et al., 2001), leading to what was termed “maladaptive cross-modal plasticity” (Bavelier & Neville, 2002; Giraud & Lee, 2007; Kral & Sharma, 2012; Lee et al., 2001). Support for this claim comes from developmental research that reports exposure to a spoken language alone contributes to better spoken language outcomes post-implantation than simultaneous exposure to both a spoken and a signed language (Geers et al., 2017; Kirk et al., 2002; Osberger & Fisher, 2000). In Osberger and Fisher’s (2000) study, deaf children whose families used a spoken language only (*n* = 40) outperformed deaf children with exposure to both a spoken and a signed language (*n* = 17) during speech perception tasks following their use of CIs for 12 months (M_AgeCI_ = 5.4 yrs). In a study with children who received their implants between two and four years of age, Kirk et al. (2002) found that deaf children in a bimodal (i.e., signed and spoken) language group (*n* = 32), as compared to a spoken language-only group (*n* = 40) showed the same rate of development for receptive language. However, only children who were exposed to a spoken language showed better results in expressive language development after three years of CI use. After analyzing a sample of 131 children (aged 8–9 yrs) four years post-implantation (M_AgeCI_ = 3.6 yrs), Tobey et al. (2004) reported that children who had been immersed in spoken communication achieved (*n* = 74) better speech intelligibility results than children who communicated using both a spoken and signed language (*n* = 57). Similarly, in a study with children who received their implants by 38 months of age, Geers et al. (2017) reported that children who only used a spoken language (*n* = 35) showed better speech recognition skills and developed speech that is more intelligible to hearing listeners after three years of CI use compared to children with exposure to both a spoken and a signed language (*n* = 62). It is important to point out that in these studies, the primary variable of interest was speech intelligibility and spoken language production rather than participants’ overall linguistic abilities. Additionally, some studies (e.g., Geers et al., 2017) categorized sign language exposure as exposure to a very broad range of communication systems, including American Sign Language (ASL), baby sign, sign support, and Pidgin sign, and all studies excluded any language measures in ASL—the child’s other language.

#### Early exposure to a signed language facilitates spoken language outcomes

Contrary to the above findings, other studies have reported better language outcomes across multiple linguistic domains for children who are exposed to a signed language in combination with a spoken language during CI use. Several studies have found benefits of exposure to signed language in educational settings that focus on developing language using both spoken and signed language. For example, Connor et al. (2000) reported that deaf children with CI in the signed and spoken language group (*n* = 66, M_AgeCI_ = 5.7 yrs) achieved significantly higher receptive spoken vocabulary scores than children in the oral (spoken) language only group (*n* = 81, M_AgeCI_ = 5.2 yrs). However, the higher scores were limited to the deaf children who received their CI before the age of five. Jiménez et al. (2009) reported better speech intelligibility scores for children in the oral/spoken communication group but superior skills in terms of verbal fluency for bimodal bilingual (sign + speech and ASL + English, respectively) children (*n* = 18, M_AgeCI_ = 3.2 yrs). In Tomasuolo et al. ’s (2010) study, children 6–14 years old attending a bilingual school (*n* = 15) (with both an oral/spoken language only and a spoken language + signed language approach) performed significantly better in picture naming tasks than the children who attended regular schools (*n* = 15) with an oral/spoken language only approach and with the support of a teaching assistant. After comparing deaf native signers and deaf children born to hearing parents and who had no experience with a signed language early in life, Hassanzadeh (2012) found that deaf children (M_age_ = 8.8 yrs) who were exposed to a signed language early in life (*n* = 7) exhibit better language outcomes in a spoken language following CI implantation. Davidson et al. (2014) reported that native signing CI users (*n* = 5, age = 4–6 yrs) performed within a typical range for hearing participants (*n* = 20) in a general test of English language, an expressive vocabulary measure, a test of articulation, a test of early literacy skills, and a measure of syntactic complexity. These findings lend support to the claim that early-life exposure to a signed language does not hinder spoken language acquisition but rather contributes to and may facilitate better spoken language outcomes.

It is worth highlighting that while the studies mentioned above mainly involved participants proficient in signed language, there is variation in the available data when examining the timing of their exposure to signed language while using CIs. Among the studies that reported the age of signed language exposure, only a limited subset of participants had the advantage of early exposure to signed language before receiving CIs (Davidson et al., 2014; Hassanzadeh, 2012; Tomasuolo et al., 2010) with a substantial proportion having acquired sign language later in life, often after undergoing CI implantation (Connor et al., 2000). Furthermore, the nature of signed language usage among participants is frequently reported to vary in degrees (Geers et al., 2017; Tomasuolo et al., 2010) or to be limited to educational settings (Connor et al., 2000; Tobey et al., 2004). Additionally, the ages at which CIs were received across these studies exhibit considerable variability. Hence, the marked differences in the age at which individuals were initially exposed to a native first language, whether through CIs or a signed language and the specific contexts of language use may provide a plausible explanation for the diverse range of research outcomes observed within this body of work (Mayberry, 2007). This is in line with the studies of spoken languages. In spoken language bilingualism, early exposure to two languages from an early age tends to lead to bilingual proficiency, with individuals exposed to their languages at an early age achieving language skills comparable to those of native speakers in both languages (e.g., Holowka et al., 2002; Kovelman et al., 2008). Conversely, late bilinguals who acquire a second language later in life, typically exhibit less optimal language performance in their newly acquired language (Hoff & Core, 2013; Kovelman et al., 2008). The age at which bilingual exposure begins, and to a lesser degree, the extent and context of language use influence the level of proficiency achieved (Berens et al., 2013; Hoff & Core, 2013). This acknowledgment underscores the age of exposure as the most significant factor shaping speech perception and language processing in deaf individuals. It is a complex interplay between the timing of first language input and the CI that potentially contributes to the nuanced patterns of outcomes observed across studies.

### Neuroimaging Studies on the Effect of Early Exposure to a Signed Language on Deaf CI Users’ Language Development

The existing body of neuroimaging studies exploring the impact of exposure to a signed language on spoken language processing in CI users has yielded mixed results. These studies have traditionally centered on the absence or reduction of sound-evoked input during early developmental stages, particularly in pre- and post-lingually deaf individuals. Historically, a prevailing theory suggested that sound deprivation might lead to maladaptive cross-modal plasticity (Giraud & Lee, 2007; Lee et al., 2001). In the context of deafness, maladaptive cross-modal plasticity was posited to occur when the visual and somatosensory systems compensated for a perceived impaired or weakened auditory sensory system (Fine et al., 2005; Finney et al., 2003). Consequently, prelingually deaf children who experienced auditory deprivation during the critical period of maximal auditory plasticity (around the age of ≈3.5 yrs; Sharma et al., 2002) and/or were exposed to sign language early in life were reported to exhibit limited improvement in hearing function even after successful CI implantation and rehabilitation (Giraud & Lee, 2007; Lee et al., 2001). Some supporting studies argued that deaf CI users engaged additional cortical areas, including auditory cortical regions, alongside visual cortical areas to process visual information, while hearing individuals primarily activated visual cortical areas. This was often interpreted as an indicator of maladaptive cross-modal plasticity (Campbell & Sharma, 2016).

However, recent research and evolving perspectives challenge this traditional viewpoint, suggesting a more balanced and nuanced understanding. Contrary to previous concerns about potential interference between visual sign language and auditory processing via CI, emerging studies have highlighted a different perspective. This updated discourse centers on the idea that signed language, as a visual modality, may not necessarily hinder the development of crucial auditory skills in CI users (Cardin et al., 2016; Corina et al., 2017; Paul et al., 2022; see Land et al., 2016, for animals). Instead, as speech inherently is multimodal (i.e., auditory speech signal is accompanied by visual of mouth movements), incorporating signed language alongside CI use may serve as a vital bridge between different sensory experiences, enhancing comprehension and communication.

Studies have shown that specific auditory regions, such as the posterior auditory field and dorsal auditory cortex, play a role in enhanced visual abilities, while the primary auditory field A1 and the anterior auditory field (AAF) are not involved, suggesting that the brain’s capacity to process auditory language remains intact even after early exposure to visual language (see Kral & Sharma, 2023, for a review). In fact, recent findings have even suggested that early exposure to visual language, especially in the case of prelingually deaf children, can have a positive impact by optimizing the restoration of hearing following implantation by leveraging both auditory and visual speech (Anderson et al., 2017). Proficiency with CIs is closely linked to the activation of early visual brain areas, and this specialization enhances as individuals become more adept at using their CIs (Rouger et al., 2007). In contrast, activation in the primary auditory cortex tends to intensify with prolonged CI use but may not necessarily become more selective in processing specific auditory stimuli (Giraud, Price, Graham, Truy, & Frackowiak, 2001). These findings suggest a mutually reinforcing relationship between auditory and visual modalities when processing the speech signal delivered by CI (Giraud, Price, Graham, & Frackowiak, 2001; Giraud, Price, Graham, Truy, & Frackowiak, 2001; Rouger et al., 2007; Strelnikov et al., 2013). In essence, early exposure to signed language can bridge the gap created by delayed CI implantation and support language acquisition and comprehension, even when auditory input remains less than optimal (Stevenson et al., 2017; Strelnikov et al., 2009). Even individuals who have experienced postlingual deafness and have previously acquired spoken language tend to rely significantly on visual cues to enhance CI performance (Rouger et al., 2007; Rouger et al., 2008). Thus, early exposure to signed language alongside CI use may serve as a powerful tool to support language development in deaf CI users, ultimately contributing to improved language outcomes (Neville et al., 1998; Strelnikov et al., 2013). Indeed, research consistently shows that those who are exposed to a language early in life, irrespective of modality, show better language outcomes (e.g., Baker et al., 2008; MacSweeney et al., 2008; Mayberry et al., 2002). Taken together, evidence from these studies suggests variable spoken language outcomes in signed language-exposed deaf CI users may not be due to maladaptive cross-modal plasticity as previously thought but rather due to the age when the deaf person was first exposed to a language.

It is also important to consider that differences in research methods and techniques applied in the previous neuroimaging studies. For example, although electroencephalography/event-related potential techniques can inform temporal patterns of activation, they provide limited information about the anatomical localization and neural systems of the underlying neural activation. Unlike these techniques, fNIRS allows us to safely measure patterns of neural activation in CI individuals by providing a noninvasive assessment of changes in blood oxygenation concentration involving oxy- and deoxy-hemoglobin (HbO and HbR), separately—all in real-time (see methods below for a more detailed description; see also Shalinsky et al., 2009, for a review). With a spatial resolution afforded through fNIRS’ optical topography technology, typically estimated at up to ∼4 cm (Shalinsky et al., 2009), fNIRS enables us to localize brain responses to specific cortical regions with reasonable precision, enhancing our understanding of the neural mechanisms involved in speech perception and language processing in deaf CI users. However, it is important to acknowledge that fNIRS, like any neuroimaging technique, has its limitations. It may not provide the same level of cortical precision as more advanced techniques like functional magnetic resonance imaging (fMRI). Notably, fMRI is not feasible with CIs due to magnetic field interactions. Therefore, while our study contributes valuable insights into the broader neural mechanisms at play, we recognize that achieving highly specific cortical localization may require complementary approaches.

### Current Study

In this study, we specifically sought to understand how exposure to a signed language at different ages interacts with the age of implantation to impact the involvement of classic left-hemisphere language networks during spoken language phonemic discrimination in deaf CI users. We especially sought adult deaf CI users who were exposed to a signed and a spoken (via CI) language at different ages. Much of the existing research on deaf individuals, including those who use a CI, has focused on group-level comparisons of individuals who were exposed to each of their languages (i.e., spoken language via CI or a signed language) either early or late. Although such research can establish fundamental group differences, it remains insensitive to the variable effects of age of language exposure on language abilities in both the signed and spoken language. In reality, bimodal CI users can rarely be classified into such discrete groups; a multitude of factors, such as family background and hearing history, contribute to the variability in the age of the exposure to each language (e.g., DeLuca et al., 2019). As a specific design feature, deaf CI users in this study received their implants and were exposed to a signed language at different ages. This design feature ensured our sample was representative of this population, and, crucially, by examining the age of language exposure effects along a continuum, this design allowed us to investigate how interactions between the age of exposure to language (ASL and English via CI) affects patterns of neural activation underlying spoken language processing.

We test three specific hypotheses concerning the impact of age of ASL exposure on behavioral performance and neural activation during phonemic discrimination in CI users who were implanted at different ages.

#### Hypothesis 1

Early exposure to a signed language has no impact (neither behavioral nor neural) on phonemic discrimination in a spoken language. Under this hypothesis, we predict no significant association between the age of ASL exposure and phonemic discrimination (*d*′ scores for native vs. non-native condition) or underlying neural activation (native vs. non-native and linguistic vs. nonlingustic conditions).

#### Hypothesis 2

Early exposure to a signed language has a positive impact (behavioral and neural) on phonemic discrimination in a spoken language. Under this hypothesis, we predict that earlier age of ASL exposure will be associated with better phonemic discrimination (increased *d*′ scores for native vs. non-native condition) and increased neural activation in left STG and IFG (native versus the non-native, and linguistic versus non-linguistic conditions).

#### Hypothesis 3

Early exposure to a signed language, regardless of the age of CI, has a negative impact (both behavioral and neural) on phonemic discrimination in a spoken language. According to this hypothesis, we predict that earlier age of ASL exposure will be consistently associated with poorer phonemic discrimination (indicated by decreased *d*′ prime scores for native versus the non-native condition) and decreased neural activation in LSTG and LIFG (native vs. non-native and linguistic vs. nonlinguistic conditions).

We further explored the influence of the age of CI and its interaction with the age of ASL exposure on phonemic discrimination and its neural correlates. While our primary hypotheses focus on the direct impact of early ASL exposure on both behavioral and neural outcomes, we probed how the age of CI implantation related to phonemic discrimination and whether the impact of ASL exposure on behavioral performance and neural activation varied depending on the age at which CI was implanted. By examining this interaction, we aimed to gain deeper insights into the diversity of outcomes observed among individuals with different combinations of ASL exposure and CI ages.

By testing these hypotheses, we aim to contribute to the evolving perspectives concerning the role of exposure to a signed language while using CI in the development of spoken language skills. In doing so, we can gain novel scientific insight into the impact of cross-modal language experience on the neural systems underlying language development, specifically on phonological processing (phonemic discrimination) so central to human language capacity, language learning, and reading. We further gain a better understanding of the impact of exposure to a signed language on the development of a spoken language for deaf and hard-of-hearing children who use a CI with potential implications for parents, clinicians, and educators to effectively support children’s language development.

## MATERIALS AND METHODS

### Participants

Eighteen deaf individuals (6 male, *M* = 20.44, *SD* = 2.1; range = 18–24 yrs, left-handed = 2) with CIs who were exposed to both a spoken (English) and signed (ASL) language at different ages participated in the study. Eighteen individuals (6 male, *M* = 18.7, *SD* = 1.06, range = 18–21 yrs, left-handed = 3) who were native monolingual hearing (H) speakers of English and who used English throughout their life participated as a comparison group.

The deaf CI users in the current study are representative of a typical bimodal CI user population that reflects the heterogeneity in terms of age of language exposure (CI and ASL) and experience with CIs. The deaf CI users received their implants between the ages of 2 and 21 years (*M* = 8.5, *SD* = 6.3) and were exposed to ASL between birth and 22 years of age (*M* = 8.6, *SD* = 7.6). The earliest age the participants in this study received their implant is around 2 to 3 years, while most of the participants were exposed to a signed language before the age of 3 years. It is important to note that although the US Food and Drug Administration now approves 9 months as the age for cochlear implantation, this was not the norm when the current adult deaf CI users first received their implants, who were in their early 20s in 2013 and 2014, when the data for the study were collected (Varadarajan et al., 2021). This explains the late age of CI implantation in some of the participants in our sample.

All CI participants reported being profoundly deaf at the time of testing (see supplementary Table 1 in the supporting information, available at https://doi.org/10.1162/nol_a_00143). Twelve participants reported using hearing aids prior to receiving their implants (*M* = 3.83; range = 1–9 yrs), and two participants reported they still use their hearing aids occasionally; however, they did not use their hearing aids during testing. All but three participants had unilateral implants (left side CI = 6, right side CI = 5, unknown = 4). We did not ask the bilateral CI users to turn off one of their CIs during testing to ensure that their CI use during testing was congruent to their day-to-day CI use for spoken language perception. This allowed obtaining a measure of their true spoken language abilities.

All participants reported their parents’ native language as spoken English, and none of the parents had a history of hearing loss. All but two participants who had early exposure to signed language (age ≤ 3 yrs) received 50% or more signed language exposure from at least one of their primary caregivers. Indeed, the quantity of ASL exposure in the first five years of life was correlated with age of exposure, indicating that those who were exposed to ASL early tended to have a primary caregiver who used ASL more frequently during this critical period and to have greater interaction with ASL-using peers. More detailed information about the quantity of ASL exposure, including details regarding the percentage and context of exposure during different life stages and the frequency of ASL use, is summarized in supplementary Table 2.

All except for three participants were born deaf. These three participants lost their hearing at a later age (*M* = 5, range = 2–8 yrs) and reported their hearing loss to be due to one of the following medical conditions: ear infection, autoimmune disease, or large vestibular aqueduct syndrome (LVAS). The deaf CI participants were all college students at Gallaudet University, where the language of instruction is signed language (ASL). The hearing participants were all college students at the University of Delaware.

Participants with neurological problems (stroke, loss of consciousness, head injury, epilepsy, coma) and/or were diagnosed with ADD or ADHD, learning disability, dyslexia or other language impairment, or Usher’s syndrome were excluded. Informed consent was obtained from all the participants, and the study received ethical approval from the institutional review boards of both Gallaudet University and University of Delaware.

### Apparatus

Data were collected at two sites: CI individuals were tested at Gallaudet University using Hitachi ETG-4000 fNIRS neuroimaging system. Monolingual English speakers were tested at University of Delaware using NIRx NIRSPORT2 fNIRS neuroimaging system. Both highly comparable neuroimaging systems, which operate on continuous wavelengths, measure the same hemodynamic responses by using noninvasive near-infrared light and measuring changes in scattered light intensity at the scalp, driven by time-varying levels of blood oxygen in the brain. We recorded the hemodynamic response with 44 channels, a standard and previously tested configuration (Plichta et al., 2007; Sato et al., 1999). The 16 sources and 14 detectors were positioned to 3 × 5 arrays corresponding to 30 probes. Wavelengths in Hitachi ETG-4000 were factory set to 695 nm and 830 nm acquiring data at 10 Hz, and wavelengths in NIRSPORT2 were factory set to 760 nm and 850 nm acquiring data at 7.4 Hz. Importantly, each system’s recording was converted to HbO and HbR changes based on system-specific wavelengths using the Beer–Lambert equation, rendering the data comparable. Previous empirical findings and modeling results show that both wavelength pairs provide converging results in the measurement of the HbO and HbR changes induced by cortical activation (Sato et al., 2004; Strangman et al., 2003). Importantly, data collected on different systems were not directly compared (see Data Processing and Analysis section).

**Figure 1.**
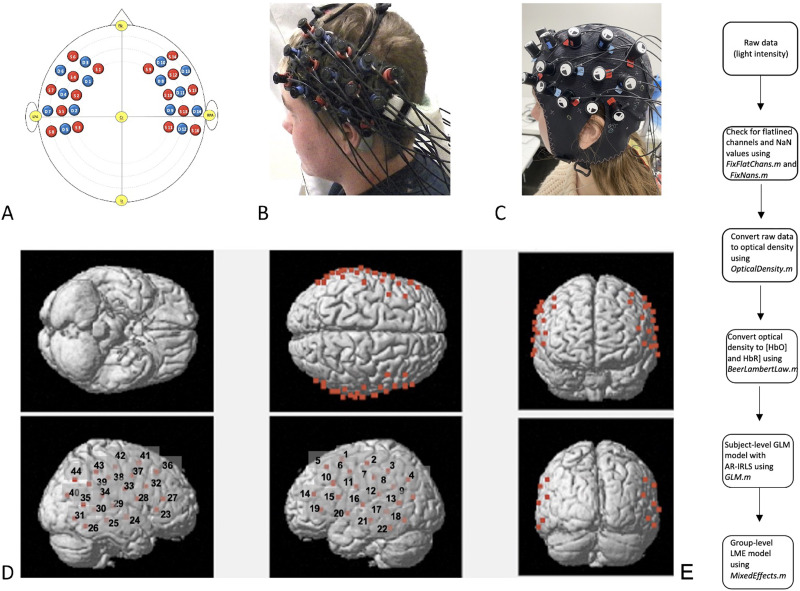
Example of functional near-infrared spectroscopy (fNIRS) placement and analysis pipeline. A. Key locations of optodes in Jasper’s (1958) 10–20 system. B. Hitachi ETG-4000 probes. C. NIRx NIRSPORT2 probes. D. Location of 44 channels. Probe arrays were placed over left-hemisphere language areas and their right-hemisphere homologs using Jasper’s 10–20 system according to the criteria shown. E. Data analysis pipeline using Brain AnalyzIR toolbox (Santosa et al., 2018). NaN = not a number, HbO = oxygenated hemoglobin response, HbR = deoxygenated hemoglobin response, GLM = generalized linear model, AR-IRLS = autoregressive, iterative robust least-squares, LME = linear mixed effects.

As is standardized in fNIRS neuroimaging, we used the 10–20 system (Jasper, 1958) to position the arrays in neuroanatomically accurate locations in order to cover classic language areas both in the left and right hemispheres (see Figure 1).

### Stimuli

Auditory stimuli consisted of three phonetic categories previously experimentally tested and published (e.g., Baker et al., 2006): (1) native English phonetic units, (2) non-native Hindi phonetic units, and (3) nonlinguistic pure tones. The native English phonetic units consisted of [ba] and [da], recorded by a male native English speaker. The non-native Hindi phonetic units consisted of [ta] (dental t) and [:ta] (retroflex t), recorded by a male native Hindi speaker (from Werker & Tees, 1984). The selection of these specific native and non-native phonemic contrasts is grounded in existing research for studying the impact of first language exposure on speech perception during infancy. These contrasts have consistently been employed to investigate perceptual narrowing in English monolingual and bilingual infants, as evidenced by previous research (Petitto et al., 2012; Werker & Hensch, 2015; Werker & Tees, 1984). Importantly, the non-native contrasts chosen for this study do not exist in English, the participants’ native language. Therefore, participants are not expected to demonstrate discrimination for these contrasts compared to contrasts present in their native English language. We used these specific contrasts to explore whether CI users who have been exposed to a language from an early age exhibit differential neural responses when discriminating between phonemes from their native spoken language compared to phonemes from a non-native language.

The nonlinguistic condition consisted of a 250 Hz pure tone. The tone condition was used to examine the participants’ hemodynamic response to linguistic (native language condition) versus nonlinguistic (tone) stimuli. All three stimuli were equated for amplitude (89 dB) and sampling rate (22 kHz), with the English and Hindi conditions also being equated for prosody and pitch.

### Procedure

While undergoing fNIRS neuroimaging, participants performed an auditory phonemic discrimination task using an oddball paradigm presented on a computer monitor using the E-Prime 3.0 software (Psychology Software Tools, 2016) and Psychopy software (Peirce et al., 2019). Participants sat at a distance of approximately 65 cm from a 22-inch monitor/speakers. Speakers placed 30 cm on each side of the monitor.

The paradigm employed a block design to ensure the reliable detection of changes in brain activity associated with each condition (native English, non-native Hindi, and non-linguistic tone). Participants were presented with 3 blocks (40 trials per block) for each linguistic condition (120 English trials and 120 Hindi trials) and 1 block (40 trials) for the nonlinguistic (tone) condition. A total of 280 trials per subject were presented. In English and Hindi conditions, one of the two phonemes was selected as a standard phoneme, while the other was selected as the target phoneme for each block. To habituate the participants to the standard phonemes, 60% of the phoneme trials were standard phonemes (24 trials), and 10% were the target phonemes (4 trials) in each block. In tasks that involve repetitive stimuli, the individuals might develop an ability to predict stimuli, leading to diminished hemodynamic activation (Fiebach et al., 2005). To reduce this anticipatory effect, “silent” trials (30%, 12 trials) were used, in which no phonemes were presented.

**Figure 2.**
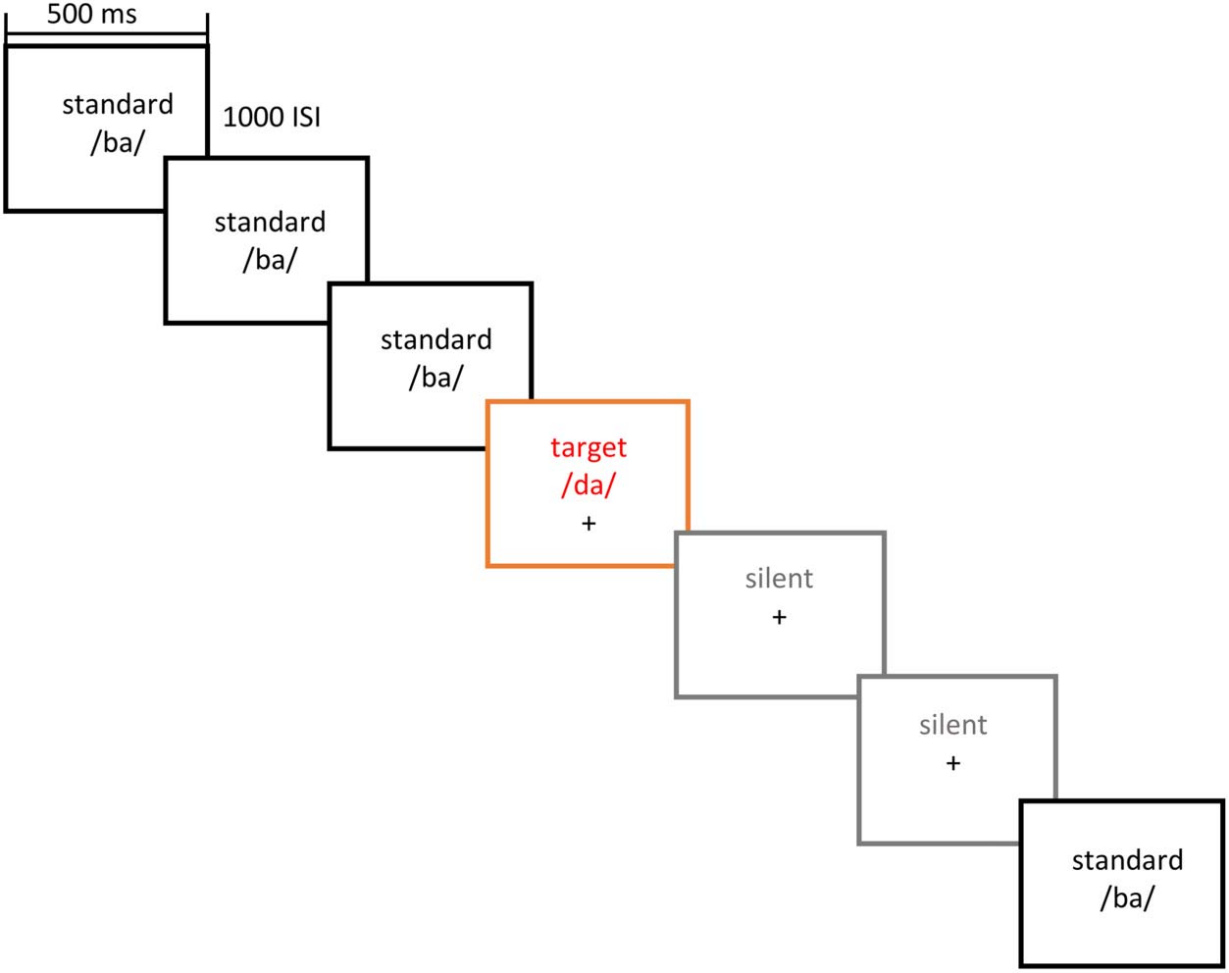
Sample run orders for auditory stimuli. Stimuli were presented for 500 ms with an interstimulus interval (ISI) of 1,000 ms.

Each phoneme served as standard and target an equal number of times. For the tone condition, 65% of the trials were tone trials (26 trials), and 35% were silent trials (14 trials). As the tone condition was used to examine the participants’ hemodynamic response to linguistic (English) versus nonlinguistic (tone) units, it did not include target and standard tones. Thus, each English and Hindi block consisted of standard, target, and silent trials, and each tone block consisted of tone trials and silent trials. We randomized the trials in each block by presenting a target trial only after a minimum of three standard trials and by having no more than two silent trials occur sequentially. Each block was 60 s long, with each stimulus lasting 500 ms with an interstimulus interval (ISI) of 1,000 ms. There was a 15 s rest between blocks (see Figure 2). The rest period was implemented to allow the stimulus-evoked hemodynamic response to revert to the baseline, a standard approach in fNIRS and fMRI block design experiments (Luke et al., 2021). Participants were told that they would hear a stream of syllables (standard trials) and were instructed to press a button upon hearing a different syllable (target trials) in English and Hindi conditions. Participants did not respond during the tone condition.

### Data Processing and Analysis

#### Behavioral analysis

To achieve accurate performance on an auditory phonemic discrimination task, it is essential that the participants not only correctly identify target syllables but also accurately reject non-target syllables. The participants’ overall error rate in such a task can be influenced by their response bias. Therefore, we calculated the sensitivity index *d*′ based on the signal detection theory to assess each participant’s ability to differentiate between targets (target trials) and non-targets (standard and silent trials), taking into account and correcting for their response bias (Green & Swets, 1966). Sensitivity analysis employing signal detection theory is a widely adopted approach in both behavioral and neuroimaging studies, particularly when dealing with oddball paradigms (e.g., Kamp & Donchin, 2015; Oates et al., 2002; Rogalsky et al., 2008). Our objective was to utilize *d*′ as a metric for sensitivity, which assesses how effectively the participants can distinguish between target and non-target stimuli.

The auditory phonemic discrimination task generated four types of responses: hit (target phoneme is present, and the participant responds), miss (target phoneme is present, but the participant fails to respond), false alarm (target phoneme is not present, and the participant responds), and correct rejection (target phoneme is not present, and the participant makes no response). Using these responses, *d*′ score was calculated using the package *neuropsychology* (Version 0.3.0; Makowski, 2016) using R software (R Core Team, 2020).

First, we examined potential differences in CI users’ and hearing individuals’ performance in auditory phonemic discrimination task using a linear mixed-effects model. Fixed effects included the main effects of group (CI, H) and language condition (Hindi, English), and a Group × Condition interaction on *d*′ scores. The random intercept of subject was included as a random effect. We used *lmer* (Bates et al., 2015) and *lmerTest* packages in R (Kuznetsova et al., 2017).

Second, to test our hypotheses, we examined the impact of age of ASL exposure on CI users’ behavioral phonemic discrimination ability. Specifically, we examined how the age of ASL exposure and CI implantation interact to influence the participant’s performance in native (English) versus non-native (Hindi) conditions using the same model. Fixed effects included Condition × Age of ASL exposure (henceforth AgeASL), Condition × Age of CI implantation (henceforth AgeCI), and Condition × AgeASL and AgeCI interactions on *d*′ scores; random effects included the random intercept of subject. Following up, we used a linear regression model to test the main effects of AgeASL and AgeCI (independent variables) on *d*′ scores in English and Hindi (dependent variables), respectively. In service of hypothesis testing, a nonsignificant, significant positive, or significant negative effect of AgeASL on English *d*′ scores would align with Hypothesis 1, 2, or 3, respectively.

#### FNIRS source localization

For CI participants, we used a 3D digitizer (Polhemus Corp.) to digitize the position of each fNIRS optode and detector and key fiducial points (nasion, inion, Cz, left and right ear) for each participant, according to previously published and well-established protocols (e.g., Kovelman et al., 2014). Prior to the experiment, the fNIRS cap was placed on each participant seated in front of a 3D magnetic digitizer stylus to obtain the relative locations of five 10–20 standard positions (Jasper, 1958; see Figure 1A) and fNIRS optodes in a real-world coordinate system. The source-detector pairs were positioned at a fixed distance of 3 cm. Using NFRI functions (National Food Research Institute Reference database with MRI datasets and the template generated by averaging all MRI brains; Okamoto & Dan, 2005; Singh et al., 2005), the estimated positions of the channels were projected from the scalp to the brain and registered to the Montreal Neurological Institute (MNI) probabilistic reference system (Mazziotta et al., 2001; Tsuzuki et al., 2012). Anatomical labels were assigned to a spherical region around each projected point using Brodmann area labels from the Automated Talairach Atlas (Lancaster et al., 2000). The probabilistic registration of fNIRS probes to standard coordinate systems is of paramount importance when aiming to compare channel-wise results with other fNIRS and fMRI studies. This registration process ensures alignment with established reference frameworks and facilitates meaningful cross-study comparisons (see supplementary Table 8 for fNIRS source localization results).

Based on the source localization results, our probe array effectively covered the bilateral frontal, temporal, and temporoparietal areas (i.e., channels 13, 16, and 20 maximally overlaid the LSTG, channels 24, 30, and 35 maximally overlaid the RSTG; channels 14, 15, and 19 maximally overlaid the LIFG, and channels 23, 27, and 28 maximally overlaid the RIFG).

To ensure that probes remained in their identical and anatomically correct pre-testing placement, we took digital photographs of the left, right, front, and top views of the probe arrays positioned on the participants’ heads before and after the recording session. We used the group-level anatomic localization we obtained from CI participants to generate hearing participants’ approximate group-level anatomical localization.

#### FNIRS analysis

The data analysis pipeline is visualized in Figure 1E. We used the NIRS AnalyzIR toolbox (Santosa et al., 2018; https://github.com/huppertt/nirs-toolbox.git) to model the effects of AgeCI, AgeASL, and stimulus condition, and their respective interactions on neural activation across 44 channels. The toolbox contains several modules for preprocessing and statistical analysis at both first-level (subject-level) and higher-level models (i.e., mixed effects, analysis of variance), published in numerous recent fNIRS studies (e.g., Pasquinelli et al., 2023; Wang et al., 2022; White & Langdon, 2021). We provide the complete MATLAB script (https://github.com/jasinskalab/GEAR) describing the analyses that follow for comparability with established practices in the field.

We followed an approach that applies minimal preprocessing to the data, as recommended by Santosa et al. (2018, p. 29). Specifically, we did not include bandpass filtering or motion correction prior to analysis. This minimal approach to preprocessing relies on statistical models, specifically here autoregressive, iterative robust least-squares (AR-IRLS) described below, that are more robust to the effects of physiology (e.g., serially correlated errors) and motion-artifacts without removing these artifacts prior to analysis. This approach is advantageous for managing high false-positive rates often encountered in fNIRS data when compared to conventional methods that involve preprocessing steps like filtering and motion artifact removal before analysis (Santosa et al., 2018). We checked whether there were any flatlined channels or NaN values existing in the data using the FixFlatChans and FixNaNs modules from the toolbox. No flatlined channels or NaN values were found. Subsequently, light intensity values were converted to optical density values using the OpticalDensity module and optical density values were converted into concentration changes in HbO and HbR responses using the Beer-Lambert law in the toolbox (Cope & Delpy, 1988; Sassaroli & Fantini, 2004).

At the subject level, we used general linear regression modeling (GLM) with AR-IRLS prewhitening approach (Barker et al., 2013; see Neuroimaging Analysis models in the supporting information). The AR-IRLS model corrects several types of physiological and motion artifacts by removing serially correlated errors in the optical data (i.e., prewhitening) and iteratively excluding high-leverage data points characteristic of transient motion artifacts in the estimate of the GLM. The AR-IRLS model therefore does not require bandpass filtering or motion correction to be applied to the data.

In the first-level (subject-level) statistical models, the design matrix specified separate predictors (coded as 1) for blocks of English, Hindi, or Tone to allow us to contrast the effects of each condition, and rest periods between blocks (coded as 0) for baseline (see section 3.1 First-Level Statistical Models in Santosa et al., 2018, pp. 10–12). We convolved the design matrix with a canonical hemodynamic response function (double gamma function) with default parameters (Santosa et al., 2018, p. 12) and fit the AR-IRLS model to the HbO data in each of the 44 channels, as HbO has been found to be more reliably correlated with blood oxygen level dependent signal changes than HbR (Sato et al., 2013; Strangman et al., 2002). The first-level statistical model generated *β* coefficients for condition at each channel for each subject.

At the group level, we fit a linear mixed effects model to the first-level *β* coefficients for each condition and the between-subjects demographic variables (AgeASL, AgeCI) with a random intercept for subject (random effect) using the MixedEffects module from the toolbox, (see section 3.5 Second-Level Statistical Models in Santosa et al., 2018, p. 16). The channel-wise regression coefficients (*β*) for each predictor and interaction were calculated and compared using Student’s *t* statistic. We used language condition (English, Hindi, Tone), AgeCI, and AgeASL as the predictors (fixed effects) and channel-wise *β* (beta values from first-level for each of the 44 channels) as the outcome variable. The model was specified as beta = condition + AgeCI + AgeASL + AgeCI × AgeASL + AgeCI × Condition + AgeASL × Condition + AgeCI × AgeASL × Condition + (1|subject), according to Wilkinson-Rogers notation.

To control for multiple comparisons across channels and model terms, we used Storey’s (2002) adaptive procedure for false-discovery rate (reported as pFDR; see Chen et al., 2009, for review) as implemented for large numbers of hypothesis tests (> 100 *p* values) in the toolbox. Thus, this correction allowed us to control FDRs across all estimated parameters and channels simultaneously when reporting the group-level analysis (Santosa et al., 2018, section 2.1.3, p. 6).

To test our hypotheses, we examined the impact of age of ASL exposure on CI users’ neural response for phonemic discrimination ability. Specifically, we examined how the age of ASL exposure and CI implantation interact to influence the involvement of classic left-hemisphere language regions during spoken language phonemic discrimination in linguistic (English) versus nonlinguistic (Tone) and in native (English) versus non-native (Hindi) conditions.

In our results, we report the interactions derived from our group level linear mixed effect model, illustrating the relationship between age of language exposure (AgeASL and AgeCI) and language conditions as follows:

AgeASL × (English > Hindi)
AgeCI × (English > Hindi)
AgeASL × AgeCI × (English > Hindi)
AgeASL × (English > Tone)
AgeCI × (English > Tone)
AgeASL × AgeCI × (English > Tone)

To interpret the Condition × AgeASL × AgeCI interactions (AgeASL and AgeCI are continuous variables), we used simple slopes analysis (an appropriate statistical technique for examining interactions between continuous variables; Aiken & West, 1991) within the toolbox, where effects are interpreted at 1 *SD* lower than the mean age for earlier language exposure (CI:2.3; ASL:1) and 1 *SD* higher than the mean age for later language exposure (CI:14.8; ASL:16.2). Through this analysis, we were able to assess and compare the influence of AgeASL on the HbO responses in each condition for individuals with early and late ages of CI implantation. In service of hypothesis testing, this analysis delves into how age of ASL exposure interacts with early versus late age of CI to impact neural activation patterns related to spoken phonemic discrimination, specifically within regions integral to phonological processing (STG, IFG). The hypothesized outcomes—whether characterized by no significant neural activation, increased neural activation, or decreased neural activation—align with Hypotheses 1, 2, or 3, respectively.

For monolingual English speakers, our model only examined the main effect of language condition (English, Hindi, Tone; beta = condition + (1|subject)), with the contrasts English > Hindi, and English > Tone.

Results were visualized using NIRS-KIT toolbox (Hou et al., 2021). We included a time course plot of HbO and HbR responses to demonstrate the presence of cortical activity above the baseline level of noise (see supplementary materials). Driven by the hypothesized involvement of the LSTG, we included a time course plot of channel 20 as a representative plot, as it emerged as a consistent finding across both groups (see supplementary materials).

To assess correlations between hemodynamic responses and behavioral performance, we conducted a correlation analysis within the toolbox, considering all 44 channels. Specifically, we examined the correlation between channel-wise *β* (from 44 channels) for native (English) versus non-native (Hindi) language contrast and behavioral *d*′ scores for English across all subjects. Storey’s (2002) adaptive FDR correction was used to control for multiple comparisons in this analysis.

## RESULTS

### Behavioral Results

**Figure 3.**
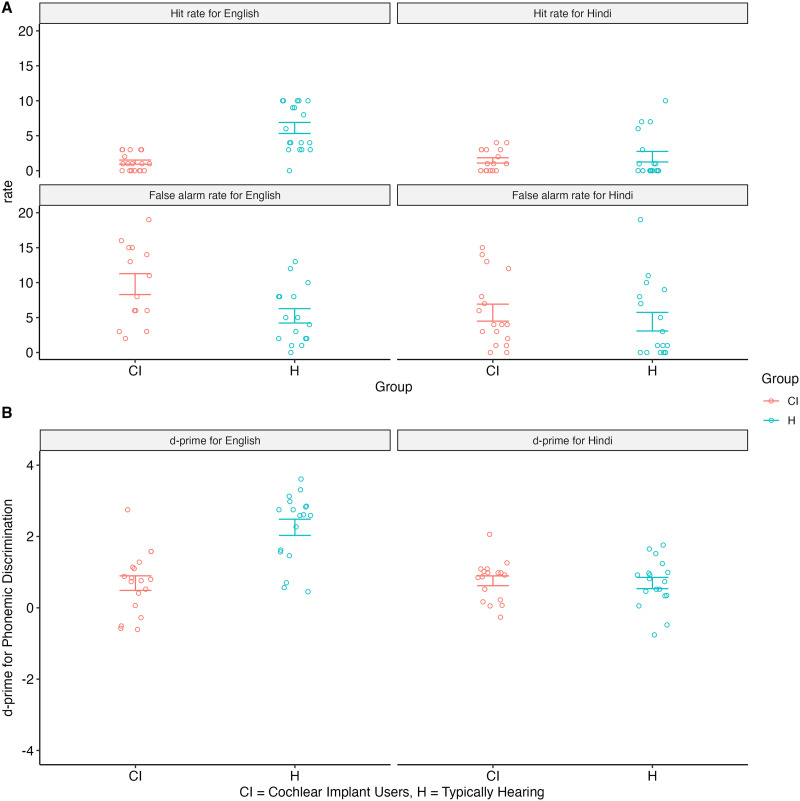
Effect of group and language and a group by language interaction on *d*′ scores using a mixed-effect model with random intercept for subject. A. Hit and False Alarm responses in each condition for each group. B. Scores (*d*′) for each condition for each group. CI = cochlear implant, H = hearing.

We first examined the effect of group and language and a group by language interaction on *d*′ scores using a mixed-effect model with random intercept for subject. We found a significant main effect of language (English vs. Hindi) but no significant main effect of group (CI vs. Hearing) on *d*′ scores (Table 1A). In general, hearing participants had higher *d*′ scores (*t*(59) = −3.33, *p* = 0.001; H: *M* = 1.48, *SD* = 1.14; CI: *M* = 0.72, *SD* = 0.73; see Figure 3 and supplementary Table 3). We also found a significant interaction between group and language on *d*′ scores (*b* = −1.62, *t*(34) = −4.537, *p* < 0.001). For the hearing group, *d*′ scores were higher for English than Hindi, *t*(30) = −5.65, *p* < 0.001 (English: *M* = 2.25, *SD* = 0.96; Hindi: *M* = 0.69, *SD* = 0.67). For the CI group, there was no significant difference between English and Hindi (English: *M* = 0.69, *SD* = 0.87; Hindi: *M* = 0.76, *SD* = 0.57); however, their mean *d*′ scores were statistically different from chance (English: *t*(17) = 3.39, *p* = 0.003; Hindi: *t*(16) = 5.51, *p* < 0.001).

**Table 1.** Behavioral Results

A. Main effect of language (English vs. Hindi) and group (CI vs. Hearing)				
Model: *d*′ score = Group + Language + Group × Language + (1|Participant)				
Predictors	*β*(*SE*)	*df*	*t*	*p*
Group [CI vs. Hearing]	0.062(0.265)	66	0.235	0.815
Language [English vs. Hindi]	1.562(0.252)	33	−6.207	4.82e−7
Group [CI vs. Hearing] × Language [English vs. Hindi]	−1.627(0.359)	34	−4.537	−6.73e−5
Language [English vs. Hindi] for Hearing	1.562(0.255)	17	6.129	1.11e−5
Language [English vs. Hindi] for CI	−.0.065(0.249)	17	−0.260	0.797
B. CI group				
Model: *d*′ score = Language × AgeCI × AgeASL + (1|Participant)				
Predictors	*β*(*SE*)	*t*(27)	*p*	
Language [English vs. Hindi]	0.193(0.606)	0.319	0.752	
Age_CI_	−0.027(0.039)	−0.654	0.519	
Age_ASL_	0.024(0.034)	0.705	0.487	
Language × Age_CI_	−0.069(0.055)	−1.265	0.217	
Language × Age_ASL_	−0.025(0.048)	−0.524	0.605	
Age_CI_ × Age_ASL_	0.002(0.003)	−0.528	0.602	
Language × Age_CI_ × Age_ASL_	0.008(0.005)	1.772	0.088	
Predictors	*β*(*SE*)	*t*(14)	*p*	
Condition = English				
Age_CI_	−0.095(0.044)	−2.221	0.044	
Age_ASL_	−0.001(0.038)	−0.030	0.976	
Age_CI_ × Age_ASL_	0.006(0.004)	1.772	0.098	
Condition = Hindi				
Age_CI_	−0.026(0.034)	−0.764	0.458	
Age_ASL_	0.024(0.029)	0.824	0.425	
Age_CI_ × Age_ASL_	−0.002(0.003)	−0.618	0.547	

*Note*. CI = cochlear implant, AgeCI = age of CI implantation, AgeASL = age of exposure to American Sign Language.

Next, we examined the effect of Language, AgeCI, AgeASL, and their respective interactions on *d*′ scores for the CI group only (see Table 1B). Although not statistically significant, we found a marginal three-way interaction between Language, AgeCI, and AgeASL on *d*′ scores (*b* = 0.008, *t*(27) = 1.772, *p* = 0.088). To further understand these effects, we conducted a two-way interaction at each level of language condition. We found a significant simple effect of AgeCI (*b* = −0.095, *t*(14) = −2.221, *p* = 0.043) for English but not Hindi, suggesting that individuals who received their CI at a younger age showed marginally higher *d*′ scores in their native language. There was no effect of AgeASL on *d*′ scores for either of the languages. Note, we tested for correlations between the AgeCI and AgeASL; there was neither a linear (*r*(34) = −0.108, *p* = 0.530) nor a quadratic relationship between these variables (*r*(34) = −0.048, *p* = 0.717).

Given that there was variability in terms of the quantity of hearing and ASL exposure, CI type, use of hearing aid, and implantation site, we conducted follow-up analyses using the same models detailed above, including additional predictors. Follow-up analyses showed that the reported results were not influenced by the inclusion of participants who were not born deaf, differences between participants with unilateral or bilateral CI, variability in the age of hearing (defined as the age of first hearing via a hearing aid or CI), quantity of ASL exposure, or the side of implantation (see supplementary materials).

### Functional NIRS Results

We examined whether there were differences or similarities in neural activation patterns for native (English) versus non-native (Hindi) language and linguistic (English) versus non-linguistic (tone) processing in English ASL–CI users and monolingual English-speaking hearing individuals. Further, we examined whether neural activation patterns differed depending on the CI individuals’ age of exposure to a signed language and the age of CI implantation. In addition, we examined the correlation between neural activation patterns and behavioral results for phonemic discrimination in native versus non-native language conditions.

#### Hearing group

We found a main effect of language condition in bilateral temporal and left frontal regions. Monolingual English speakers showed a significantly increased activation in the left hemisphere language areas, including in the channels overlaying the bilateral temporal lobes (L: channels 8, 9, 13, 20, 22; R: 31, 40, 44) and left frontal regions (channels 10, 19), but decreased activation in the left inferior frontal region (channel 15) and right temporal region (channel 29) for English versus Hindi condition (Table 2A). In the English versus tone condition, there was significant bilateral activation in channels overlaying the superior middle and posterior temporal gyri (L: channels 8, 9, 13, 18, 20, 21; R: channels 24, 30, 31, 40, 44) as well as the prefrontal cortex (channels 10, 19, 32) (Table 2B). We present a representative time course plot of HbO and HbR responses in LSTG for speech compared to the baseline in the supplementary materials.

**Table 2.** Hearing monolingual speakers’ neural activation patterns (HbO)

Channel	Approximate location	*β*(*SE*)	*t*(52)	pFDR
A. English vs. Hindi				
44	R SMG of Wernicke’s	26.072(3.438)	7.584	1.00e−7
20	Anterior L STG	20.704(3.287)	6.299	3.05e−6
40	R AG of Wernicke’s	14.371(2.427)	5.921	7.46e−6
5	L Frontal eye fields	20.059(4.445)	4.508	4.11e−4
31	Posterior R MTG	19.460(2.506)	7.764	7.42e−8
9	L AG of Wernicke’s	14.397(2.755)	5.225	8.11e−6
8	L SMG of Wernicke’s	20.925(4.071)	5.140	6.61e−5
10	L DLPFC	13.039(3.489)	3.737	0.002
13	Posterior L STG	9.904(2.731)	3.627	0.003
22	Posterior L MTG	11.783(3.585)	3.286	0.007
19	L IPFG	9.958(3.212)	3.100	0.010
15	L IFG, pars triangularis	−8.811(2.302)	−3.827	0.002
29	Anterior R STG	−7.501(2.714)	−2.763	0.023
B. English vs. Tone				
21	Posterior L MTG	38.278(3.497)	10.945	3.84e−13
8	L SMG of Wernicke’s	37.519(5.827)	6.439	2.22e−7
31	Posterior R MTG	37.203(3.626)	10.261	1.94e−12
10	L DLPFC	31.697(4.794)	6.612	1.44e−7
44	R SMG of Wernicke’s	31.407(4.812)	6.527	1.71e−7
40	R AG of Wernicke’s	29.813(3.488)	8.548	3.21e−10
9	L AG of Wernicke’s	29.751(3.984)	7.467	1.67e−8
18	Posterior L MTG	29.074(5.027)	5.784	1.68e−6
20	Anterior L STG	24.113(4.669)	5.164	1.26e−5
13	Posterior L STG	17.120(3.859)	4.437	1.25e−4
24	Anterior R STG	20.212(5.163)	3.915	5.87e−4
36	R Frontal eye fields	19.009(6.758)	2.813	0.009
30	Posterior R STG	11.777(3.951)	2.981	0.006
5	L Frontal eye fields	16.822(6.305)	2.668	0.013
19	L IPFG	18.091(4.627)	3.909	0.014
32	R DLPFC	−25.254(10.606)	−2.381	0.024

*Note*. *β* indicates brain activity strength; channel numbers correspond to channel locations in Figure 1D. HbO = oxygenated hemoglobin, pFDR = Storey’s (2002) adaptive procedure for false-discovery rate, R = right, L = left, SMG = supramarginal gyrus, STG = superior temporal gyrus, AG = angular gyrus, MTG = middle temporal gyrus, DLPFC = dorsolateral prefrontal cortex, IPFG = inferior prefrontal gyrus, IFG = inferior frontal gyrus.

#### Deaf CI users group

We found main effects of AgeCI, AgeASL, and Language conditions as well as interactions between AgeCI and Language, AgeASL and Language, and AgeASL and AgeCI in bilateral temporal and frontal regions (Table 3). We also found a significant three-way interaction between language condition, AgeASL, and AgeCI in the bilateral STG, left MTG, and right IFG (channels 3, 16, 20, 22,29; Table 3; see Table 8 in the supplementary materials for MNI coordinates of each channel). Earlier age of ASL exposure was associated with neural activation in bilateral STG, LMTG, and left dorsolateral prefrontal cortex (DLPFC) during English phonemic discrimination (channels 6, 14, 20, 22, 24, 29; Table 3, English × AgeASL). Earlier age of CI implantation was associated with greater neural activation in the pars triangularis part of Broca’s area in the right hemisphere during English phonemic discrimination (channel 28; Table 3B, English × AgeCI).

**Table 3.** CI users’ neural activation patterns (HbO)

Channel	Brain Area (coverage probability)	*β*(*SE*)	*t*(36)	pFDR
A. English vs. Hindi				
	*English*			
40	R AG of Wernicke’s (0.74)	−13.551(4.595)	−2.949	0.091
	*Age*_*CI*_			
	No significant activation			
	*Age*_*ASL*_			
23	R IPFG (0.35)	1.494(0.438)	3.409	0.044
	*Age_CI_ × Age_ASL_*			
3	L SMG of Wernicke’s (0.66)	−0.406(0.105)	−3.871	0.018
23	R IPFG (0.35)	−0.235(0.068)	−3.456	0.040
	*English × Age_CI_*			
	No significant interaction			
	*English × Age_ASL_*			
29	Anterior R STG (0.35)	−2.524(0.558)	−4.521	0.005
6	L DLPFC (0.39)	2.851(0.683)	4.176	0.009
	*English × Age_CI_ × Age_ASL_*			
23	R IPFG (0.35)	0.292(0.086)	3.403	0.044
16	Anterior L STG (0.48)	0.504(0.164)	3.072	0.075
B. English vs. Tone				
	*English*			
3	L SMG of Wernicke’s (0.66)	20.231(3.370)	6.004	1.28e−4
35	Posterior R STG (0.79)	13.595(2.453)	5.542	3.73e−4
28	R IFG, pars triangularis (0.57)	12.690(2.807)	4.522	0.003
	*Age*_*CI*_			
22	Posterior L MTG (0.69)	−2.489 (0.775)	−3.213	0.053
	*Age*_*ASL*_			
24	Anterior R STG (0.62)	2.864(0.594)	4.820	0.002
20	Anterior L STG (0.59)	1.782(0.451)	3.945	0.011
13	Posterior L STG (0.80)	1.612(0.444)	3.636	0.021
	*Age_CI_ × Age_ASL_*			
29	Anterior R STG (0.35)	0.412(0.097)	4.244	0.007
	*English × Age_CI_*			
28	R IFG, pars triangularis (0.57)	−2.137(0.588)	−3.636	0.021
	*English × Age_ASL_*			
24	Anterior R STG (0.62)	−3.331(0.603)	−5.524	3.73e−4
22	Posterior L MTG (0.69)	−2.504(0.616)	−4.064	0.010
20	Anterior L STG (0.59)	−1.685(0.468)	−3.601	0.023
14	L DLPFC (0.52)	−1.684(0.470)	−3.579	0.024
	*English × Age_CI_ × Age_ASL_*			
20	Anterior L STG (0.59)	−0.325(0.085)	−3.811	0.016
29	Anterior R STG (0.35)	−0.449(0.094)	−4.742	0.002
22	Posterior L MTG (0.69)	0.363(0.114)	3.184	0.056

*Note*. *β* indicates brain activity strength. Using a simple-slopes analysis to compare the simple effects of AgeASL and AgeCI for English versus Hindi condition, we found that when AgeCI was early (1 *SD* below mean age; age = 2.3 yrs), no significant effects of AgeASL on neural activation in classic language areas were observed (all |betas| < 2.73, pFDR > 0.10). By contrast, when AgeCI was late (1 *SD* above mean age; age = 14.6 yrs), earlier AgeASL was associated with increased activation in anterior L STG (channel 20) but decreased activation in frontal regions (channel 23; Table 4A). CI = cochlear implant, HbO = oxygenated hemoglobin, pFDR = Storey’s (2002) adaptive procedure for false-discovery rate, R = right, L = left, AgeCI = age of CI implantation, AgeASL = age of exposure to American Sign Language, AG = angular gyrus, IPFG = inferior prefrontal gyrus, SMG = supramarginal gyrus, STG = superior temporal gyrus, DLPFC = dorsolateral prefrontal cortex, MTG = middle temporal gyrus, IFG = inferior frontal gyrus.

**Figure 4.**
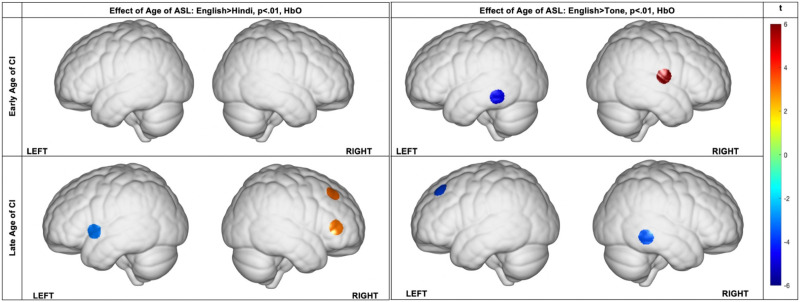
Simple slopes channel-wise HbO regression coefficient maps depicting the *t* score (*t*) for each condition. Maps show the effect of age of ASL exposure × Task for CI users with early and later age of CI implantation, for English versus Hindi and English versus Tone conditions, respectively.

In the English versus tone condition, we found that when AgeCI was early, earlier AgeASL was associated with increased activation in posterior LMTG (channel 22) but decreased activation in more anteriorly located RSTG (channel 29). By contrast, when AgeCI was late, earlier AgeASL was associated with increased activation in posterior RMTG (channel 26) and frontal eye fields (Table 4B; see also Figure 4 for the brain plots).

**Table 4.** Simple slope effects of AgeASL × Task for early and late ages of AgeCI

Channel	Brain area (coverage probability)	MNI coordinates			*β*(*SE*) Age_ASL_ × Condition	*t*(23)	pFDR
		*x*	*y*	*z*			
*A. English vs. Hindi*							
	*Early Age_CI_*						
	No significant effect				|*β*| < 0.273	|*t*| < 3.625	>0.010
	*Late Age_CI_*						
20	Anterior L STG (0.59)	−60	8.33	−3	−2.587(0.838)	−3.087	0.069
23	R IPFG (0.35)	57.33	37.33	4.33	3.204(1.043)	3.071	0.069
36	R Frontal eye fields (0.71)	41.333	33.33	46.33	3.253(1.008)	3.227	0.069
*B. English vs. Tone*							
	*Early Age_CI_*						
22	Posterior L MTG (0.69)	−69	−42.33	−14.67	−5.346(1.141)	−4.686	0.004
29	Anterior R STG (0.35)	70	−10.33	11.67	4.879(0.648)	7.529	1.04e−5
	*Late Age_CI_*						
26	Posterior R MTG (0.71)	70.33	−43.67	−11.33	−1.253(0.356)	−3.516	0.067
5	L Frontal eye fields (0.62)	−35.33	37.33	45.33	−1.149(0.311)	−3.695	0.067

*Note*. *β* indicates change in difference English > (Hindi or Tone) with varying ages of ASL exposure. AgeASL = age of exposure to American Sign Language, AgeCI = age of CI implantation, pFDR = Storey’s (2002) adaptive procedure for false-discovery rate, L = left, R = right, STG = superior temporal gyrus, IPFG = inferior prefrontal gyrus, MTG = middle temporal gyrus.

### Brain–Behavior Correlations

We performed correlation analyses to examine the relationships between behavioral *d*′ scores and hemodynamic responses (from 44 channels) in both the hearing group and the deaf CI users’ group. The correlation was conducted between hemodynamic responses in English versus Hindi and *d*′ scores for English. The specific channels displaying significant correlations are detailed in Table 5.

**Table 5.** Brain–behavior correlations (HbO)

Channel	Approximate location		*r*		*t*(52)		pFDR
A. Hearing monolingual speakers’ brain–behavior correlations							
5	L Frontal eye fields		0.729		7.685		<0.01
21	Posterior L MTG		0.723		7.543		<0.01
3	L SMG of Wernicke’s		0.691		6.897		<0.01
40	R AG of Wernicke’s		0.536		4.577		<0.01
44	R SMG of Wernicke’s		0.523		4.428		<0.01
9	L AG of Wernicke’s		0.450		3.625		<0.01
17	Anterior L STG		−0.456		−3.692		<0.01
26	Posterior R MTG		−0.699		−7.054		<0.01
B. CI users’ brain–behavior correlations							
Channel	Brain area (coverage probability)	MNI Coordinates			*r*	*t*(45)	pFDR
		x	y	z			
3	L SMG of Wernicke’s	−63	−33	48	0.528	4.170	<0.05
25	Posterior R STG	69	−48.33	16.33	0.438	3.266	<.1

*Note*. HbO = oxygenated hemoglobin, pFDR = Storey’s (2002) adaptive procedure for false-discovery rate, L = left, R = right, MTG = middle temporal gyrus, SMG = supramarginal gyrus, AG = angular gyrus, STG = superior temporal gyrus, CI = cochlear implant.

In the hearing group, higher *d*′ scores were positively correlated with activation in the anterior and middle temporal lobe and posterior temporoparietal and frontal regions; negative correlations were observed in the right posterior middle temporal lobe and left anterior superior temporal lobe (Table 5A). In the deaf CI users group, higher *d*′ scores for English phonemic discrimination were positively correlated with neural activation in bilateral temporal regions (RSTG and LSMG; Table 5B).

## DISCUSSION

In this study, we examined patterns of neural activation underlying phonemic discrimination in deaf individuals using CIs who were exposed to a signed language at different ages, as compared to lifelong hearing English speakers. We sought to better understand the long-term impact of exposure to a signed language on the development of a spoken language for deaf and hard-of-hearing children who use a CI with potential implications for parents, clinicians, and educators to support children’s language development effectively.

Although deaf CI users, in general, showed poorer performance on the phonemic discrimination task compared to hearing individuals, the deaf CI users who received their CI earlier in life discriminated native language phonemes better than deaf CI users who received their CI later in life. Moreover, we observed no effects of ASL exposure on phonemic discrimination ability. Intriguingly, differences in neural activation patterns of deaf CI users with earlier age of exposure to a signed language as compared to deaf CI users with later age of exposure to a signed language provide potential evidence for the positive impact of early signed language acquisition in supporting deaf CI users’ spoken language development, in support of Hypothesis 2. However, these findings should be interpreted cautiously, given that our observations are based on a small number of deaf CI users, many of whom were exposed to signed language later in life.

### Phonemic Discrimination in Hearing Individuals and Deaf CI Users

Behavioral results revealed that, overall, and as expected, hearing participants discriminated phonemes better in the native language as compared to the non-native language condition. Deaf CI users with early age of CI implantation demonstrated slightly better phonemic discrimination in native language over deaf CI users who received their implant later in life. It is important to note that most deaf CI users in our study received their CI at or after three years of age. The age of implantation has been argued to be a strong predictor of auditory perception and development of a spoken language in prelingually deaf individuals who receive their implants at an age younger than three years (Hassanzadeh et al., 2002). Our results show that deaf CI users’ ability to discriminate phonemes in a spoken language still lags behind that of hearing individuals. Importantly, this is true even for individuals who received a CI and or hearing aids at a young age and who use their CI regularly.

Furthermore, the concept of early implantation in the context of cochlear implants has evolved over time, and it is essential to acknowledge and discuss this evolution in the context of our study. In the early years of cochlear implantation, the age at which individuals in this study received their CIs was often considerably later than what is considered early intervention today. Historically, the field lacked the diagnostic tools and awareness to identify hearing loss in infants and provide timely intervention. Recent advancements in newborn hearing screening and early diagnosis have enabled CIs to be performed in the first year of life or even within the first few months, aligning with research emphasizing the significance of early language exposure. Our study, focusing on adults who received cochlear implants at ages 2 or 3 years, acknowledges the changing landscape of cochlear implantation and language exposure practices. We recognize that, in many cases, our participants may not have had access to language exposure equivalent to those who received implants at younger ages in today’s context. This lack of early language exposure is a significant factor that may have influenced their language development and phonemic discrimination skills.

The poor performance of deaf individuals with CIs on the phonemic discrimination task may also be, in part, attributed to the poorer spectral resolution of consonant contrasts delivered through CIs due to a smaller range of audible frequencies and a small number of frequency channels (Loizou et al., 2009) as compared with the auditory system of hearing individuals. Previous studies have shown that even though some deaf individuals who use CIs succeed in discriminating fine-grained temporal cues (i.e., small changes to voice onset time) at a level similar to hearing individuals (Caldwell & Nittrouer, 2013), they perform poorly in discriminating consonants based on their place of articulation, which requires fine-grained spectral cues (Giezen et al., 2010). For example, Xu et al. (2005) showed that even though both types of cues are important for phoneme recognition, within the category of consonants, spectral cues were more important than temporal cues to distinguish sounds based on place of articulation. This could explain deaf CI users’ poor performance in discriminating /b/ and /d/ sounds, as the only distinguishing feature between these consonants is the place of articulation.

Another important factor to note is that the majority of our participants, except for three, had a unilateral implant. Although our comparisons of results with and without bilateral implant users included did not yield substantive differences in our participants’ behavioral performance, access to bilateral auditory cues, such as differences in intensity of the signals and time of arrival of signals reaching the two ears, makes it possible to localize sound sources and understand speech in typical listening situations (Kühn-Inacker et al., 2004). Deaf individuals using CIs with unilateral implants who have no access to these bilateral cues are almost certain to have difficulties in detecting and processing auditory information successfully (Sarant et al., 2014). Being restricted to monaural hearing input and, thus, being deprived of the advantages of binaural and spatial hearing may affect deaf CI users’ performance negatively on all such tasks.

Although we did not find a significant relationship between age of ASL exposure and phonemic discrimination abilities in spoken language, it is important to note the lack of a negative effect of ASL on spoken phonemic discrimination skills via CI. The finding may suggest that ASL exposure, regardless of age, does not hinder or impede individuals with a CI from developing phonemic discrimination abilities in spoken English. This indicates that individuals who are exposed to ASL, even at a later age, can still benefit from CI intervention for spoken language processing without negative consequences on their spoken phonemic discrimination skills. However, it is crucial to approach this finding with due caution. We acknowledge that the absence of a significant effect in our study does not definitively rule out the potential influence of ASL on spoken phonemic discrimination abilities; instead, it highlights the need for further research to replicate the existing findings and explore this aspect comprehensively.

### Neural Correlates of Phonemic Discrimination in Hearing Individuals and Deaf CI Users

The neural correlates of phonological processing in deaf CI users with earlier age of exposure to a signed language provided evidence in support of Hypothesis 2. Early life language exposure, irrespective of modality, involves the recruitment of specific language regions in the left temporal cortex, with the select involvement of corresponding tissue on the right. Overall, deaf individuals using CIs with an earlier age of exposure to a signed language showed significant left-lateralized activation. Specifically, deaf individuals using CIs with an earlier age of exposure to a signed language showed significantly greater involvement of LSTG, RSTG, LMTG, and left DLPFC for English phonemic discrimination (Table 3; English × AgeASL); in particular, activity in bilateral temporal regions was positively correlated with phonemic discrimination in both hearing and deaf individuals.

#### Native versus non-native language

In the native versus non-native language condition, hearing individuals showed significantly greater activation, chiefly in the bilateral temporoparietal regions (Table 2). Notably, their neural responses in bilateral temporoparietal regions were positively associated with their behavioral performance (Table 5A).

Deaf individuals with CIs also showed significantly greater activation in bilateral supramarginal gyrus (Wernicke’s area) to native versus non-native language conditions (Table 3). Their neural responses in the left supramarginal gyrus (Wernicke’s area) and posterior RSTG were positively correlated with their behavioral performance (Table 5B). However, it is crucial to approach the interpretation of correlations cautiously, particularly when making comparisons between hearing individuals and those with CI. The CI users exhibited notably weaker correlations, emphasizing the need for careful consideration and interpretation of results in this specific group.

Importantly, we observed different effects of age of ASL on neural activation in bilateral temporal and frontal regions depending on the age at which deaf individuals received a CI. For deaf individuals who received a CI early, there was no effect of age of ASL exposure on brain activation (Table 4A and Figure 4). That is, simultaneous CI and ASL use did not impede brain activation canonically associated with phonemic processing. However, for deaf individuals who received a CI later in life, earlier ASL exposure was associated with increased activation in the LSTG but decreased activation in frontal regions (RIPFG, frontal eye fields). The intriguing pattern of neural activation resonates with established fNIRS activity patterns found in individuals with early and late acquisition of second languages where it has been observed that the age of exposure can modify neural mechanisms that support language processing (see Cargnelutti et al., 2019, for review; Jasińska & Petitto, 2013).

In previous research, it has been consistently observed that early-exposed proficient bilinguals exhibit greater activation in LSTG compared to later-exposed or lower-proficiency bilinguals (Cargnelutti et al., 2019; Jasińska & Petitto, 2013). This effect is attributed to the constant demands of dual-language co-activation from an early age and enhanced language skills (Chee et al., 2003; Kovelman et al., 2009). Whereas late-exposed bilinguals tend to exhibit greater neural response in frontal regions that are attributed to increased attention and inhibition control (Abutalebi & Green, 2016; Liu & Cao, 2016). Similarly, late CI users with delayed ASL exposure might experience cognitive demands that prompt heightened activation in these frontal areas, potentially enhancing attention and inhibiting interference during language processing. Thus, this pattern of neural activation in our cohort appears to resonate with neural activation typically associated with language processing demands in later-exposed or lower-proficiency bilinguals rather than a maladaptive response. For individuals who received their CI later in life and had earlier exposure to ASL, the heightened activation in the LSTG may indicate improved phonemic discrimination abilities, potentially resulting in enhanced speech perception and comprehension. This enhanced neural response in the LSTG may be driven by early exposure to ASL. Meanwhile, the observed reduction in frontal activation could suggest more efficient neural processing, where fewer cognitive resources are allocated to phonemic discrimination due to increased proficiency. This interpretation aligns with the notion that early exposure to a language, whether spoken or signed, may aid the brain’s neural architecture to optimize language processing efficiency.

#### Linguistic versus non-linguistic

In the linguistic versus non-linguistic conditions, here again, we observed the important effects of age of ASL on neural activation. For deaf individuals who received a CI early, early exposure to ASL was associated with greater activation in posterior LMTG and less activation in the anterior RSTG (Table 4B and Figure 4). This significant neural activation increase in LMTG for linguistic alternating phonemes versus non-linguistic tones was predicted as the left temporal cortical areas are specialized for the analysis of rapidly changing information (Hickok & Poeppel, 2004; Zatorre & Belin, 2001). Many previous studies report activation in the left temporal lobe, including broader areas around STG and MTG when listening to speech stimuli versus resting baseline or non-speech stimuli (Ashtari et al., 2004; Vouloumanos et al., 2001). This greater activation in posterior LMTG was observed in hearing participants as well. Furthermore, in the hearing group, this heightened activation in the LMTG was positively correlated with their behavioral performance, underlining a robust brain–behavior relationship (Table 5A), although we did not observe a significant correlation between LMTG activation and *d*′ scores in the CI group.

We also observed increased activation in RSTG in CI users with early age of CI implantation, with this effect strengthening as the age of ASL exposure increased. Notably, this pattern was consistent in brain–behavior correlations, where the neural response in the RSTG (albeit this was observed more posteriorly) exhibited a positive association with behavioral performance. However, it is important to highlight that in both the hearing and CI groups, the brain–behavior correlations in bilateral temporoparietal cortices were strongest in the left hemisphere.

This observation carries important implications for our understanding of language processing in this population. In considering the greater RSTG activation in CI users with early age of CI and late age of ASL, it is informative to draw parallels with the findings from early and late bilingualism studies. Studies in bilingual individuals have consistently demonstrated that late bilinguals tend to exhibit greater neural activation in the right hemisphere, particularly in the right anterior temporal lobe (Bavelier & Neville, 2002; Jasińska & Petitto, 2013; Mitsuhashi et al., 2020). This increased activation may be linked to the unique cognitive demands associated with processing a second language acquired later in life. Additionally, the RSTG has been identified as a crucial region for language switching and is associated with the attentional demands of language processing (Luk et al., 2012). These parallels further suggest that right hemisphere involvement may reflect distinct processing demands in individuals with later language exposure. Specifically, the greater involvement of RSTG in CI users with late ASL exposure compared to those with early ASL exposure may indicate increased language processing demands, and the weaker involvement of RSTG in CI users with early ASL exposure may possibly be indicative of less effortful neural processing.

It is essential to acknowledge that while these findings provide valuable insights into the differences in neural processing between individuals with normal hearing and those with CIs, there are inherent distinctions in their language experiences. Thus, caution should be exercised when extrapolating findings from the hearing group to the CI group, as the observed variations may reflect the complex interactions between sensory and linguistic experiences (Bedny et al., 2015; Cargnelutti et al., 2019; Jasińska & Petitto, 2013).

### Language Deprivation Hypothesis

Our behavioral results corroborate previous studies examining spoken language processing in deaf CI populations that showed that the majority of CI users underperform in language comprehension and expression tasks when compared to hearing individuals (e.g., Niparko et al., 2010). A pervasive explanation for such findings has been attributed to the fact that implants offer a generally reduced quality of speech input that can be perceived through a cochlear implant as compared to the human ear (Zeng et al., 2008).

Here, we offer a different view. Together with the fNIRS data, we suggest that the deaf CI users’ performance may be related to the conditions of *language deprivation* that some deaf CI users experienced prior to receiving their implants and/or hearing aids. Receiving no other early life experience with an accessible and natural human language, such as a signed language, prior to implantation and/or hearing aids causes them to have *no language input* (*zero*) during vital and key sensitive periods universal to language development (Cheng et al., 2018; Emmorey, 2018; Hall et al., 2019; Woll, 2018). Regardless of modality, early exposure to any language builds a strong linguistic foundation for language learning. Our view is in line with Hall et al. ’s (2019) language scaffolding hypothesis, according to which having no access to any linguistic input early in life leads to both social and cognitive impairments, and thus, to reduce the effects of language deprivation, deaf children should have access to a natural signed language early in their development (Hall et al., 2019).

The present behavioral findings revealed that (i) early age of CI implantation positively impacts phonemic discrimination in a spoken language and (ii) early exposure to a signed language does not impact phonemic discrimination in a spoken language. Importantly, the neural findings revealed that the age of exposure to a natural first language is crucial for the development of language processing across different modalities. We witnessed that the earlier age of exposure to a signed language showed robust involvement of the same classic language tissue recruitment as in hearing individuals when processing the phonemic discrimination task in English (bilateral temporoparietal regions). We, therefore, hypothesize that the deficits that we see in the spoken language outcomes of deaf individuals using CIs are, at least in part, due to language deprivation; the lack of early exposure to a natural first language such as signed language, which can serve as a foundation for the development of a spoken language (Hall, 2017; Hall et al., 2019; Skotara et al., 2012).

What we observe in our results is that the same language function, phonological processing, occurs in the same brain sites and systems in all participants who were exposed to a natural first language, irrespective of modality. Surprisingly, we found no conclusive evidence of neural disruption when a deaf CI user was exposed to a signed language early—prior to or at the same age they received their hearing aids or CIs. We suggest that it is not the modality of the input that is key in development (auditory vs. signed), but rather the *age* at which the individuals were exposed to a natural first language that affects the deaf CI users’ later language outcomes.

However, it is crucial to acknowledge that the interpretation of our fNIRS and behavioral data regarding the effects of early ASL exposure on phonemic discrimination in a spoken language is open to different interpretations that warrant cautious consideration. For example, additional variation in the quantity and quality of early life language exposure may further contribute to phonemic discrimination abilities and may be an important avenue for future research. Nonetheless, we found that the reported quantity of early-life ASL exposure was relatively consistent in participants who were exposed to ASL early, underscoring the importance of age, rather than amount, of language exposure in language outcomes. We encourage a cautious approach to interpreting our results, recognizing the complexity of factors at play in the development of language skills among this unique population. Our study contributes to the ongoing exploration of these complex dynamics and their impact on language outcomes in individuals with CIs and early ASL exposure.

### Limitations

While our findings provide evidence for the impact of age of signed language exposure in CI users’ spoken language processing, some limitations of this study must be noted. First, a notable limitation of our study is the relatively small sample size. Our participant cohort comprised 18 adults who are both ASL and CI users. While our research sought to examine the complex interplay between the ages of exposure to ASL and CI and their influence on spoken phonemic discrimination skills and neural activation patterns, the modest sample size may restrict the broader applicability of our findings. However, a significant strength of our study is that all participants are fluent users of both signed and spoken languages via CI. While the population of fluent deaf signers and population of CI users are notably larger, our study sample is drawn from a relatively small population of fluent deaf signers who also use a CI. We acknowledge this constraint and encourage readers to interpret our results in light of this limitation.

Second, given that our CI participants were exposed to both a signed and a spoken language at different ages, an ideal comparison hearing group would have consisted of normally hearing age-matched bilingual speakers, including bimodal bilinguals (e.g., hearing children of deaf adults), who were exposed to their languages at different ages. Exposure to two languages in early life has the potential to modify neural activation patterns underlying the ability to process language in the left-hemisphere language areas and their right-hemisphere homologues (Gernsbacher & Kaschak, 2003; Kovelman et al., 2008; Kovelman et al., 2014; Price, 2012); however, there are crucial similarities between monolingual and bilingual individuals’ language processing. For example, research has shown that monolinguals and bilinguals recruit the same language-specific brain areas, such as LSTG and LIFG, for phonological and syntactic processing, with bilinguals showing a greater extent of recruitment of the LIFG (Jasińska & Petitto, 2013, 2018). Future research would benefit from comparing bilingual CI individuals and unimodal/bimodal bilingual hearing individuals.

Third, the specific nature, type, and contexts in which these participants received their early language input vary among participants. In the past, the language therapy offered after implantation was not necessarily obligatory, and unfortunately, several CI recipients might not have had access to an appropriate language input early on and, therefore, might not have reached their full potential with their CIs. This might be one of the reasons why some of our participants reported using their CIs occasionally. Another important factor is that the participants in this study used ASL in addition to CI for social, academic, and cultural purposes, as evidenced by the fact that they were attending a university where the language of instruction is a signed language. This suggests that their proficiency in ASL allows full participation in university in ASL (e.g., sufficient proficiency to attend and participate in lectures, meet with faculty, conduct group work). This also explains why almost all of the participants (16 out of 18) reported using ASL all the time. This might be another reason why some deaf CI users were not using their CI regularly despite getting their CI early in life.

In the same vein, it is possible that the occasional CI users use their implants mostly for sound awareness and not for language processing. This could be due to receiving CIs late and/or having other impairments at the level of the central auditory system. In addition, the majority of our participants had unilateral implants, which are common for adult deaf CI users who were in their early 20s in 2013–2014, when the data for the study were collected. Bilateral cochlear implantation was not the standard of care for children with severe to profound hearing loss until the early 2000s (Varadarajan et al., 2021). It is possible that having bilateral implants might yield different outcomes. It has been reported that children with bilateral CIs achieve significantly better spoken language outcomes than children with unilateral CIs (Sarant et al., 2014). The impact of exposure to a signed language on spoken language processing in deaf CI users with bilateral implants has to be further investigated.

Finally, we cannot rule out the possibility that the neural activation patterns we observed in our results are related to the gender imbalance in our current sample (6 males in each group). Evidence from some neuroimaging studies indicates that lateralization difference exists between genders, with males showing more left-lateralized activation and females showing more bilateral activation during language tasks (Clements et al., 2006). However, results are inconsistent, especially in the studies that involved auditory phonological and comprehension tasks (Sommer et al., 2004). Future studies would benefit from exploring whether gender differences are apparent in the population of CI users.

### Implications

There has been a long-standing belief that exposure to a non-auditory modality language, such as sign language, hinders the development of spoken language skills in deaf individuals using CIs. Despite limited evidence, recent findings suggest a shift in this perspective, indicating that deaf CI users who learn sign language from an early age can achieve comparable spoken language outcomes to their hearing peers (e.g., Davidson et al., 2014; Kral & Sharma, 2023).

When sign language exposure is delayed, withheld, and/or not fully accessible, as is typically the practice used with deaf CI users, there is a possibility that these deaf individuals using CIs will not display typical language processing neural circuitry. These neural changes can be misinterpreted as a negative impact of being exposed to a signed language. They are more likely a result of language deprivation during vital developmental periods of language acquisition. Based on these misinterpretations, in most cases, parents and caregivers choose not to expose their deaf children to fully accessible visual signed language prior to cochlear implantation. This approach usually leads to a considerably more burdensome process of learning a spoken language (post-implantation), as children do not have a pre-existing linguistic foundation to build on. When considering between a signed plus a spoken language and a spoken language alone, parents and caregivers should be made aware that exposure to a natural first language, such as a signed language, may facilitate the development of post-implant spoken language skills. In order to effectively support deaf CI users’ healthy language development, language interventions should prioritize the full acquisition of a natural first-language foundation (signed language) rather than exclusively focusing on an auditory input-only approach only once a child receives a CI.

## CONCLUSION

The present study is among only a few to combine the investigation of deaf individuals using CI at both the behavioral and neural levels. We explored how exposure to two languages (English via CI and a signed language, i.e., ASL) across a continuum of different ages impacts language processing and its underlying brain activation patterns. Using fNIRS neuroimaging optical topography uniquely suited to the study of individuals with electrical-based CI technology, this work revealed novel insights about the differential levels of cortical activation during a phonological processing task.

One crucial finding of our study is that being exposed to a signed language early in life does not appear to negatively impact the neural underpinnings of language processing in a spoken language. Here, we suggested that differential spoken language outcomes among deaf individuals using CIs are not due to auditory deprivation but might rather be linked to the age at which the individual was initially exposed to a natural first language. This is corroborated by the observation of the effect of early life exposure to a signed language on neural activations in the left hemisphere’s classic language regions. In many cases, caregivers of deaf children are discouraged from using a signed language in fear that it might impede the development of a spoken language post-implantation. Our findings suggest that exposure to a natural signed language in very young children prior to CI implantation does not prevent subsequent development of a spoken language using a CI and may indeed facilitate it.

## ACKNOWLEDGMENTS

We extend our heartfelt thanks to individuals from the Gallaudet University and the University of Delaware communities for their participation. We thank Gallaudet University BL2’s research assistants and the University of Delaware’s BOLD lab research assistants for helping with data collection. We also thank Janet Werker for sharing her pioneering stimuli with us.

## FUNDING INFORMATION

Laura-Ann Petitto, National Institute of Health, Award ID: NIHR01HD04582203. Laura-Ann Petitto, National Science Foundation (https://dx.doi.org/10.13039/100000001), Award ID: SBE1041725.

## AUTHOR CONTRIBUTIONS

**Shakhlo Nematova**: Conceptualization: Equal; Data curation: Equal; Formal analysis: Lead; Investigation: Lead; Methodology: Equal; Project administration: Lead; Software: Lead; Validation: Equal; Visualization: Lead; Writing – original draft: Lead; Writing – review & editing: Equal. **Benjamin Zinszer**: Formal analysis: Supporting; Validation: Supporting; Visualization: Supporting; Writing – review & editing: Equal. **Thierry Morlet**: Writing – review & editing: Equal. **Giovanna Morini**: Writing – review & editing: Equal. **Laura-Ann Petitto**: Conceptualization: Lead; Funding acquisition: Lead; Methodology: Lead; Resources: Lead; Validation: Equal; Writing – review & editing: Equal. **Kaja K. Jasińska**: Conceptualization: Lead; Formal analysis: Equal; Investigation: Lead; Methodology: Lead; Project administration: Lead; Resources: Lead; Software: Equal; Supervision: Lead; Validation: Lead; Visualization: Supporting; Writing – original draft: Supporting; Writing – review & editing: Lead.

## DATA AND CODE AVAILABILITY STATEMENT

All original source code for behavioral and neuroimaging analyses, as well as the behavioral data, are available at https://github.com/jasinskalab/GEAR. Neuroimaging data are available at https://osf.io/gear_nirs_data. Functional NIRS brain imaging data were converted, analyzed, and verified using the NIRS Brain AnalyzIR Toolbox (September 16, 2020) with MATLAB R2020a: https://github.com/huppertt/nirs-toolbox.git.

## Supplementary Material


