# Supplementary material for: Impact of ASL Exposure on Spoken Phonemic Discrimination in Adult CI Users: A Functional Near-Infrared Spectroscopy Study
Source: Neurobiol Lang (Camb). 2024 Jun 14;5(2):553–88. doi: 10.1162/nol_a_00143 (PMC11210937; doi:10.1162/nol_a_00143)
Supplement: Supplementary file 1 [file nol-5-2-553-s001.pdf]

Table 1

Cochlear Implant users

CI users	Age	Gender	Handedness	Born deaf	Hearing loss reason/age	Hearing loss severity at time of testing	Hearing aid (HA)/ age	Age ASL	Age CI	Type of CI	Unilateral (U) or Bilateral (B) CIs	Side of CI	Use CI	Use ASL	Use HA	Years of hearing experience via CI (CI+HA)	Years of ASL experience
1	24	F	R	No	Recurring ear infections /2	Profound	No	15	3	Nucleus Freedom	U	L	regularly	all the time/daily basis	No	21	9
2	18	F	R	Yes		Profound	No	2	2	Harmony	U	R	regularly	all the time/daily basis	No	16	16
3	18	M	L	Yes		Profound	Yes/5	3	12	Nucleus Freedom (N5)	B		regularly	all the time/daily basis	No	6(13)	15
4	22	M	R	Yes		Profound	Yes/4	4	9	Nucleus Freedom	U	L	regularly	all the time/daily basis	No	13(18)	18
5	18	F	R	Yes		Profound	Yes/3	3	13	Nucleus Freedom (N5)	U	Unknown	occasionally	all the time/daily basis	occasionally	5(15)	15
6	20	M	R	No	Autoimmune disease/8	Profound	Yes/9	18	11	Nucleus Freedom (N5)	U	Unknown	regularly	all the time/daily basis	No	9(11)	2
7	19	M	R	Yes		Profound	No	0	5	Nucleus Freedom	B		occasionally	all the time/daily basis	No	14	19

8	20	F	R	Yes		Profound	Yes/3	2	13	unknown	U	Unknown	occasionally	all the time/daily basis	No	7(17)	18
9	22	M	L	Yes		Profound	Yes/7	21	9	unknown	U	L	occasionally	all the time/daily basis	No	13(15)	1
10	19	F	R	Yes		Profound	Yes/3	2	3	Harmony	U	L	occasionally	all the time/daily basis	No	16(16)	17
11	22	F	R	Yes		Profound	Yes/1	22	2.3	Nucleus 22 internal, Epsut 3G External	U	R	regularly	once/week	No	19.7(21)	0
12	18	F	R	Yes		Profound	No	17	3	Nucleus Freedom (N5)	B		regularly	few times/week	No	15	1
13	19	F	R	Yes		Profound	No	7	3	unknown	U	L	occasionally	all the time/daily basis	No	16	12
14	19	F	R	Yes		Profound	No	7	3.5	Harmony	U	R	occasionally	all the time/daily basis	No	15.5	12
15	23	F	R	No	LVAS/5	Profound	Yes/6	18	21	unknown	U	Unknown	occasionally	all the time/daily basis	No	2(17)	5
16	23	F	R	Yes		Profound	Yes/2	2	21	Nucleus Freedom	U	L	occasionally	on special occasions	occasionally	2(21)	21

17	24	F	R	Yes	Profound	Yes/3	3	9	Harmony	U	R	occasionally	all the time/daily basis	No	15(21)	21
18	20	F	R	Yes	Profound	Yes/3	3	15	Nucleus Freedom	U	R	regularly	all the time/daily basis	No	5(17)	17

Note: unknown denotes that the participant did not disclose the type of CI they used

Table 2
History of ASL exposure

	Percent exposure		Age/ Number of participants		
			birth – 5 years	5 – 17 years	18 – time of testing
Mother used ASL with the participant	80-100%		3	3	4
	50-80%		3	5	5
	20-50%		5	3	2
	1-20%		1	1	2
	0		6	6	5
Father used ASL with the participant	80-100%		1	2	2
	50-80%		3	2	2
	20-50%		2	2	2
	1-20%		1	2	2
	0		11	10	10
Participant used ASL with friends	80-100%		7	8	13
	50-80%		1	2	2
	20-50%		3	1	0
	1-20%		1	3	2
	0		5	4	1
Frequency of ASL usage at the time of testing	all the time		daily basis	few times a week	once a week
	12		3	1	2
Parents	Native language	Hearing	DHH	use ASL/Y	use ASL/N
	English	18	0	11	7

In this section, we delve into the relationships between the age at which ASL exposure began and the quantity of ASL exposure during different life stages (see Table 2). We also discuss the implications of these relationships on both behavioral and neural outcomes in CI users.

Correlations between Age of ASL Exposure and Nature of ASL Exposure: To investigate potential connections between the timing and quantity of ASL exposure, we performed Spearman correlation analyses. These analyses unveiled noteworthy associations between the age of ASL exposure and the nature of ASL exposure across various life periods. Our analysis unveiled intriguing correlations between the age of ASL exposure and the quantity of ASL exposure across various life stages. These findings provide valuable insights into the dynamics of language exposure:

First 5 Years of Life: During this crucial developmental period, we observed significant correlations between the age of ASL exposure and the quantity of ASL exposure. Specifically, we found significant correlations between age of ASL exposure and maternal ASL use (r = -0.45, p = 0.058), and ASL usage with friends (r = -0.57, p = 0.013), indicating that participants exposed to ASL earlier tended to have mothers who used ASL more frequently during this critical period and to have greater interaction with ASL-using peers. The correlation between age of ASL exposure and paternal ASL use was not significant (r = -0.39, p = 0.106). These correlations suggest that the age at which ASL exposure commenced had measurable associations with the extent of ASL interaction within families and social circles during early childhood.

Beyond the First Five Years: Our analyses also extended beyond the early years, examining age ranges from 6 to 17 and ages 18 and above. Surprisingly, we found similar patterns of correlation between age and quantity of ASL exposure in contexts during these later life stages. For ages 6-17, significant correlations were noted for maternal ASL use (r = -0.63, p = 0.005) and ASL usage with friends (r = -0.60, p = 0.008); paternal ASL use was not significant (r = -0.36, p = 0.141). For ages 18 and older, correlations persisted for maternal ASL use (r = -0.67, p = 0.002 and ASL usage with friends (r = -0.54, p = 0.022); paternal ASL use was not significant (r = -0.35, p = 0.161). This suggests that early ASL exposure may have long-term effects on ASL engagement and communication patterns.

These intriguing results collectively underscore the importance of considering the quantity of ASL exposure in addition to age of exposure as a multifaceted variable when investigating language outcomes in CI users, offering potential avenues for future research and clinical interventions.

However, we do find that those who were exposure to ASL early did report high (>50%) exposure to ASL from the primary caregiver , with relatively limited within-group (i.e. those with early age of ASL exposure) variance in the quanity of input

Table 3
*d'*score, hit rate and false alarm for each language condition

Group	Participan t	English d-prime	Hindi d-prime	English hit rate	Hindi hit rate	English false alarm rate	Hindi false alarm rate
H	1	3.13	0.521	4	0	2	1
H	2	0.45	1.52	4	1	23	0
H	3	2.75	0.346	10	0	8	1
H	4	2.85	0.97	8	0	2	0
H	5	2.59	0.921	3	0	1	0
H	6	2.61	1.65	10	7	2	3
H	7	3.61	1.24	10	7	4	8
H	8	1.57	0.462	6	1	8	7
H	9	0.703	0.735	0	0	0	0
H	10	2.83	0.337	9	1	8	9
H	11	1.62	-0.48	3	0	10	11
H	12	3.31	1.76	10	3	5	0
H	13	2.98	0.993	10	6	3	10
H	14	2.75	0.521	4	0	5	1
H	15	1.46	0.054	3	0	13	5
H	16	2.27	-0.759	4	0	12	19
H	17	0.566	0.817	9	10	35	67
H	18	2.59	0.921	3	0	1	0
CI	1	0.842	2.06	1	3	14	1
CI	2	0.735	0.983	0	1	3	3
CI	3	-0.576	0.983	0	4	21	12
CI	4	1.58	1.02	3	3	6	7
CI	5	0.523	1.26	2	2	21	2
CI	6	1.1	0.921	3	0	15	0
CI	7	-0.509	1.09	0	3	19	6
CI	8	-0.277	0.07	0	1	13	13
CI	9	1.28	0.852	3	4	11	15
CI	10	0.762	0.982	1	3	16	14
CI	11	1.14	0.87	1	1	8	4
CI	12	0.879	1.09	0	0	2	0
CI	13	0.801		1		15	
CI	14	2.75	0.223	3	0	6	4
CI	15	-0.608	0.521	0	0	22	1
CI	16	1.58	0.054	3	0	6	4
CI	17	0.409	-0.262	0	0	3	8
CI	18	0.066	0.168	1	0	22	3

Given that three participants had bilateral CIs and three participants were not born deaf, we further conducted analyses excluding bilateral CI users and CI users who were not born deaf; the results from the

model that included only unilateral CI users showed that the previously marginal interaction between language, age of CI, and age of ASL on *d'* scores in the full dataset remained marginally significant (bilateral CI excluded: b=.009, t(21)=1.96, p=.063; full results are available in Supplementary materials). To understand this interaction, we conducted this analysis at each level of language condition. There was a marginal two-way interaction between the age of CI and the age of ASL for English condition only (b=.007, t(11)=2.082, p=.061). No significant effects were observed in the model that excluded CI users who were not born deaf.

Because 12 participants reported using hearing aids prior to implantation, we conducted the same analysis by replacing the age of CI with the age of hearing (defined as a first exposure to sound either via hearing aids or CI). There was no significant effect of age of hearing on *d'* scores in either of the language conditions. Finally, we examined if there is an effect of the side of implantation on *d'* scores; no significant effects of side of implantation were observed. Similar patterns of results were observed in our neuroimaging result. Age of hearing and side of implantation are therefore excluded from further models.

That the age of hearing (the age of first exposure to sound) did not predict CI users' spoken phonemic discrimination skills, neither behaviorally nor neurally is not surprising as previous research has shown that CI use was associated with greater sound and speech improvement than hearing aid use (Cohen et al., 2004). Exposure to sound with hearing aids is not similar to exposure to spoken language with CIs. Admittedly, the current study population is the subset of CI users with profound hearing loss who ultimately stopped using their hearing aids and decided to get implanted to support their spoken language processing. This fact implies that the exposure to sound via hearing aids was not sufficient to facilitate the perception of fine-grained spoken language cues for this group of CI users. Even if a cochlear implant provides sufficient acoustic information to support language processing in a spoken language, it does not restore full hearing

Table 4
Behavioral results with bilateral CI users excluded

d' score = Language*AgeCI *AgeASL + (1|Participant) *(bilateral CI users excluded)*

**Predictors**	**Estimate**	**S.E.**	**t(21)**	**p**
Language [English vs Hindi]	0.746	0.655	1.139	0.267
Age CI	-0.021	0.040	-0.530	0.602
Age ASL	0.034	0.038	0.903	0.377
Language * Age CI	-0.089	0.056	-1.576	0.130
Language * Age ASL	-0.057	0.053	-1.073	0.296
Age CI * Age ASL	-0.002	0.003	-0.622	0.541
Language * Age CI * Age ASL	0.009	0.004	1.964	**0.063**
Condition = English				
Age CI	-0.110	0.041	-2.710	**0.020**
Age ASL	-0.023	0.039	-0.591	0.566
Age CI * Age ASL	0.007	0.003	2.082	**0.061**

Table 5
Behavioral results with CI users who were not born deaf excluded

d' score = Language*AgeCI *AgeASL + (1|Participant) *(CI users who were not born deaf excluded)*

**Predictors**	**Estimate**	**S.E.**	**t(21)**	**p**
Language [English vs Hindi]	0.209	0.647	0.324	0.749
Age CI	-0.027	0.044	-0.621	0.541
Age ASL	-0.001	0.046	-0.020	0.984
Language * Age CI	-0.068	0.061	-1.100	0.284
Language * Age ASL	0.007	0.065	0.105	0.917
Age CI * Age ASL	0.001	0.007	0.174	0.863
Language * Age CI * Age ASL	0.005	0.010	0.455	0.654

Table 6
Behavioral results with CI users' age of hearing

d'score = Language*Agehearing *AgeASL + (1|Participant.ID)

**Predictors**	**Estimate**	**S.E.**	**t(27)**	**p**
Language [English vs Hindi]	1.237	1.096	1.128	0.269
Age Hearing	0.072	0.199	0.362	0.720
Age ASL	0.024	0.049	0.485	0.632
Language * Age Hearing	-0.451	0.281	-1.604	0.120
Language * Age ASL	-0.038	0.070	-0.538	0.595
Age Hearing * Age ASL	0.003	0.012	-0.255	0.800
Language * Age Hearing * Age ASL	0.022	0.017	1.335	0.193

Table 7
Behavioral results with CI users' side of implant

d'score = Language*Agehearing *AgeASL*sideCI + (1|Participant.ID)

**Predictors**	**Estimate**	**S.E.**	**t(5)**	**p**
Language [English vs. Hindi]	1.74	5.83	0.30	.778
Age ASL	1.79	1.48	1.21	.280
Age hearing	0.55	0.59	0.94	.393
side [bilateral]	-10.34	16.76	-0.62	.564
side [left]	4.87	5.71	0.85	.432
side [right]	7.64	5.04	1.51	.190
Language [English vs. Hindi] * Age ASL	-0.65	2.09	-0.31	.768
Language [English vs. Hindi] * Age hearing	-0.43	0.83	-0.52	.623
Age ASL* Age hearing	-0.20	0.17	-1.18	.290
Language [English vs. Hindi]* side [bilateral]	6.94	23.70	0.29	.781
Language [English vs. Hindi]* side [left]	-5.03	7.92	-0.63	.554
Language [English vs. Hindi]* side [right]	-1.87	7.13	-0.26	.804
Age ASL* side [bilateral]	-0.83	0.72	-1.15	.301
Age ASL* side [left]	-1.80	1.49	-1.21	.282

Age ASL* side [right]	-1.96	1.49	-1.31	.246
Age hearing* side [bilateral]	2.53	3.47	0.73	.499
Age hearing* side [left]	-0.47	1.27	-0.37	.725
Age hearing* side [right]	-1.94	1.35	-1.43	.212
Language [English vs. Hindi]*Age ASL* Age hearing	0.09	0.24	0.36	.730
Language [English vs. Hindi]* Age ASL* side [bilateral]	0.23	1.01	0.23	.830
Language [English vs. Hindi]*Age ASL* side [left]	0.86	2.11	0.41	.701
Language [English vs. Hindi]* Age ASL* side [right]	0.49	2.11	0.23	.827
Language [English vs. Hindi]* Age hearing*side [bilateral]	-1.62	4.90	-0.33	.754
Language [English vs. Hindi]* Age hearing *side [left]	1.34	1.75	0.76	.480
Language [English vs. Hindi]* Age hearing *side [right]	0.15	1.92	0.08	.941
Age ASL* Age hearing* side [left]	0.20	0.18	1.11	.316
Age ASL* Age hearing* side [right]	0.31	0.21	1.43	.213
Language [English vs. Hindi]*Age ASL *Age hearing*side [left]	-0.14	0.25	-0.54	.611
Language [English vs. Hindi] Age ASL*Age hearing*side [right]	0.11	0.30	0.36	.732

Table 8
Channel probabilistic brain MNI coordinates and Brodmann areas Automated Talairach atlas labels

			MNI Coordinates				
Channel	Source	Detector	x	y	z	Brodmann areas	Coverage probability
1	1	1	-39.33	18.33	58.33		
						6 - Pre-Motor and Supplementary Motor Cortex	0.38
						8 - Includes Frontal eye fields	0.62
2	2	1	-54.33	-7.33	53.67		
						1 - Primary Somatosensory Cortex	0.01
						3 - Primary Somatosensory Cortex	0.25

						4 - Primary Motor Cortex	0.13
						6 - Pre-Motor and Supplementary Motor Cortex	0.62
3	2	2	-63.00	-33.00	48.00		
						1 - Primary Somatosensory Cortex	0.10
						2 - Primary Somatosensory Cortex	0.22
						3 - Primary Somatosensory Cortex	0.02
						40 - Supramarginal gyrus part of Wernicke's area	0.66
4	3	2	-59.67	-57.67	39.67		
						39 - Angular gyrus, part of Wernicke's area	0.18
						40 - Supramarginal gyrus part of Wernicke's area	0.83
5	1	3	-35.33	37.33	45.33		
						8 - Includes Frontal eye fields	0.63
						9 - Dorsolateral prefrontal cortex	0.38
6	4	1	-53.00	11.33	43.33		
						6 - Pre-Motor and Supplementary Motor Cortex	0.29
						8 - Includes Frontal eye fields	0.31
						9 - Dorsolateral prefrontal cortex	0.39
7	2	4	-65.00	-14.67	38.67		
						1 - Primary Somatosensory Cortex	0.16
						2 - Primary Somatosensory Cortex	0.08
						3 - Primary Somatosensory Cortex	0.18
						4 - Primary Motor Cortex	0.07
						6 - Pre-Motor and Supplementary Motor Cortex	0.51

8	5	2	-67.00	-41.33	32.33		
						40 - Supramarginal gyrus part of Wernicke's area	1.00
9	3	5	-60.00	-65.33	22.67		
						19 - V3	0.12
						22 - Superior Temporal Gyrus	0.14
						39 - Angular gyrus, part of Wernicke's area	0.74
						40 - Supramarginal gyrus part of Wernicke's area	0.01
10	4	3	-51.00	31.33	30.33		
						9 - Dorsolateral prefrontal cortex	0.27
						45 - pars triangularis Broca's area	0.03
						46 - Dorsolateral prefrontal cortex	0.70
11	4	4	-64.00	3.33	27.33		
						4 - Primary Motor Cortex	0.03
						6 - Pre-Motor and Supplementary Motor Cortex	0.68
						9 - Dorsolateral prefrontal cortex	0.21
						44 - pars opercularis, part of Broca's area	0.04
						45 - pars triangularis Broca's area	0.04
12	5	4	-68.67	-24.33	22.00		
						1 - Primary Somatosensory Cortex	0.06
						2 - Primary Somatosensory Cortex	0.15
						22 - Superior Temporal Gyrus	0.05
						40 - Supramarginal gyrus part of Wernicke's area	0.35
						42 - Primary and Auditory Association Cortex	0.25

						43 - Subcentral area	0.15
13	5	5	-68.00	-49.33	13.33		
						21 - Middle Temporal gyrus	0.16
						22 - Superior Temporal Gyrus	0.80
						40 - Supramarginal gyrus part of Wernicke's area	0.04
14	6	3	-45.33	50.67	16.33		
						10- Frontopolar area	0.48
						46 - Dorsolateral prefrontal cortex	0.52
15	4	6	-59.00	22.33	14.67		
						44 - pars opercularis, part of Broca's area	0.25
						45 - pars triangularis Broca's area	0.70
						46 - Dorsolateral prefrontal cortex	0.05
16	7	4	-67.00	-7.00	10.00		
						4 - Primary Motor Cortex	0.02
						6 - Pre-Motor and Supplementary Motor Cortex	0.10
						21 - Middle Temporal gyrus	0.02
						22 - Superior Temporal Gyrus	0.48
						42 - Primary and Auditory Association Cortex	0.20
						43 - Subcentral area	0.18
17	5	7	-71.00	-34.33	2.33		
						21 - Middle Temporal gyrus	0.46
						22 - Superior Temporal Gyrus	0.45
						42 - Primary and Auditory Association Cortex	0.10

18	8	5	-65.33	-57.33	-4.33		
						21 - Middle Temporal gyrus	0.51
						37 - Fusiform gyrus	0.49
19	6	6	-53.33	41.67	0.33		
						10 - Frontopolar area	0.11
						45 - pars triangularis Broca's area	0.17
						46 - Dorsolateral prefrontal cortex	0.16
						47 - Inferior prefrontal gyrus	0.56
20	7	6	-60.00	8.33	-3.00		
						6 - Pre-Motor and Supplementary Motor Cortex	0.01
						21 - Middle Temporal gyrus	0.20
						22 - Superior Temporal Gyrus	0.59
						38 - Temporopolar area	0.15
						44 - pars opercularis, part of Broca's area	0.04
						45 - pars triangularis Broca's area	0.01
						47 - Inferior prefrontal gyrus	0.00
21	7	7	-71.00	-17.67	-9.67		
						20 - Inferior Temporal gyrus	0.01
						21 - Middle Temporal gyrus	0.96
						22 - Superior Temporal Gyrus	0.04

22	8	7	-69.00	-42.33	-14.67		
						20 - Inferior Temporal gyrus	0.19
						21 - Middle Temporal gyrus	0.69
						37 - Fusiform gyrus	0.11
23	9	8	57.33	37.67	4.33		
						10 - Frontopolar area	0.04
						45 - pars triangularis Broca's area	0.31
						46 - Dorsolateral prefrontal cortex	0.30
						47 - Inferior prefrontal gyrus	0.35
24	10	8	64.00	6.00	0.00		
						6 - Pre-Motor and Supplementary Motor Cortex	0.07
						21 - Middle Temporal gyrus	0.17
						22 - Superior Temporal Gyrus	0.62
						38 - Temporopolar area	0.05
						44 - pars opercularis, part of Broca's area	0.09
25	10	9	73.00	-20.67	-6.33		
						21 - Middle Temporal gyrus	0.82
						22 - Superior Temporal Gyrus	0.18
26	11	9	70.33	-43.67	-11.33		
						20 - Inferior Temporal gyrus	0.11
						21 - Middle Temporal gyrus	0.72
						37 - Fusiform gyrus	0.17
27	9	10	51.00	45.67	18.67		
						10 - Frontopolar area	0.24

						46 - Dorsolateral prefrontal cortex	0.76
28	12	8	63.00	18.33	17.67		
						9 - Dorsolateral prefrontal cortex	0.05
						44 - pars opercularis, part of Broca's area	0.37
						45 - pars triangularis Broca's area	0.57
29	10	11	70.00	-10.33	11.67		
						4 - Primary Motor Cortex	0.03
						6 - Pre-Motor and Supplementary Motor Cortex	0.01
						22 - Superior Temporal Gyrus	0.36
						40 - Supramarginal gyrus part of Wernicke's area	0.02
						42 - Primary and Auditory Association Cortex	0.30
30	13	9	73.00	-35.67	4.67		
						21 - Middle Temporal gyrus	0.22
						22 - Superior Temporal Gyrus	0.70
						42 - Primary and Auditory Association Cortex	0.09
31	11	12	67.00	-56.00	-1.00		
						21 - Middle Temporal gyrus	0.61
						37 - Fusiform gyrus	0.39
32	12	10	55.67	26.33	31.67		
						9 - Dorsolateral prefrontal cortex	0.52
						45 - pars triangularis Broca's area	0.08

						46 - Dorsolateral prefrontal cortex	0.40
33	12	11	67.00	0.33	28.67		
						3 - Primary Somatosensory Cortex	0.00
						4 - Primary Motor Cortex	0.04
						6 - Pre-Motor and Supplementary Motor Cortex	0.79
						9 - Dorsolateral prefrontal cortex	0.14
						43 - Subcentral area	0.01
						44 - pars opercularis, part of Broca's area	0.00
34	13	11	71.00	-25.67	22.67		
						1 - Primary Somatosensory Cortex	0.05
						2 - Primary Somatosensory Cortex	0.12
						22 - Superior Temporal Gyrus	0.03
						40 - Supramarginal gyrus part of Wernicke's area	0.47
						42 - Primary and Auditory Association Cortex	0.26
						43 - Subcentral area	0.07
35	13	12	69.00	-48.33	16.33		
						21 - Middle Temporal gyrus	0.05
						22 - Superior Temporal Gyrus	0.79
						40 - Supramarginal gyrus part of Wernicke's area	0.16
36	14	10	41.33	33.33	46.33		
						8 - Includes Frontal eye fields	0.72
						9 - Dorsolateral prefrontal cortex	0.28

37	12	13	57.67	8.33	43.67		
						6 - Pre-Motor and Supplementary Motor Cortex	0.52
						8 - Includes Frontal eye fields	0.20
						9 - Dorsolateral prefrontal cortex	0.28
38	15	11	67.00	-17.67	40.33		
						1 - Primary Somatosensory Cortex	0.20
						2 - Primary Somatosensory Cortex	0.13
						3 - Primary Somatosensory Cortex	0.16
						4 - Primary Motor Cortex	0.11
						6 - Pre-Motor and Supplementary Motor Cortex	0.39
						40 - Supramarginal gyrus part of Wernicke's area	0.01
39	13	14	68.00	-41.67	33.67		
						40 - Supramarginal gyrus part of Wernicke's area	1.00
40	16	12	60.67	-63.33	26.67		
						22 - Superior Temporal Gyrus	0.05
						39 - Angular gyrus, part of Wernicke's area	0.75
						40 - Supramarginal gyrus part of Wernicke's area	0.21
41	14	13	43.33	15.67	57.67		
						6 - Pre-Motor and Supplementary Motor Cortex	0.48
						8 - Includes Frontal eye fields	0.52

42	15	13	57.00	-10.33	53.67		
						1 - Primary Somatosensory Cortex	0.08
						3 - Primary Somatosensory Cortex	0.34
						4 - Primary Motor Cortex	0.13
						6 - Pre-Motor and Supplementary Motor Cortex	0.45
43	15	14	64.67	-34.67	49.33		
						1 - Primary Somatosensory Cortex	0.07
						2 - Primary Somatosensory Cortex	0.16
						40 - Supramarginal gyrus part of Wernicke's area	0.77
44	16	14	60.00	-56.67	42.67		
						39 - Angular gyrus, part of Wernicke's area	0.11
						40 - Supramarginal gyrus part of Wernicke's area	0.89

Neuroimaging models

```
% subject level GLM to estimate task-level effects
glm_job = nirs.modules.GLM;
glm_job.type = 'AR-IRLS'
SubjectLevelStats = glm_job.run(HB_data);

%LME to test across subjects
lme_job = nirs.modules.MixedEffects;
lme_job.formula='beta ~-1 + task+AgeCI+AgeASL+ AgeCI: AgeASL + AgeCI:task+ AgeASL:task+ AgeCI: AgeASL:task+(1|subject)';
lme_job.dummyCoding = 'effect';
HbModel = lme_job.run(SubjectLevelStats);
```

Figure 1

Hemodynamic response functions (HRF) for the oxygenated (HbO, red) and deoxygenated (HbR, blue) response signal in channel 20 for the English language condition in the hearing group (A; left) and the CI group (B;right)

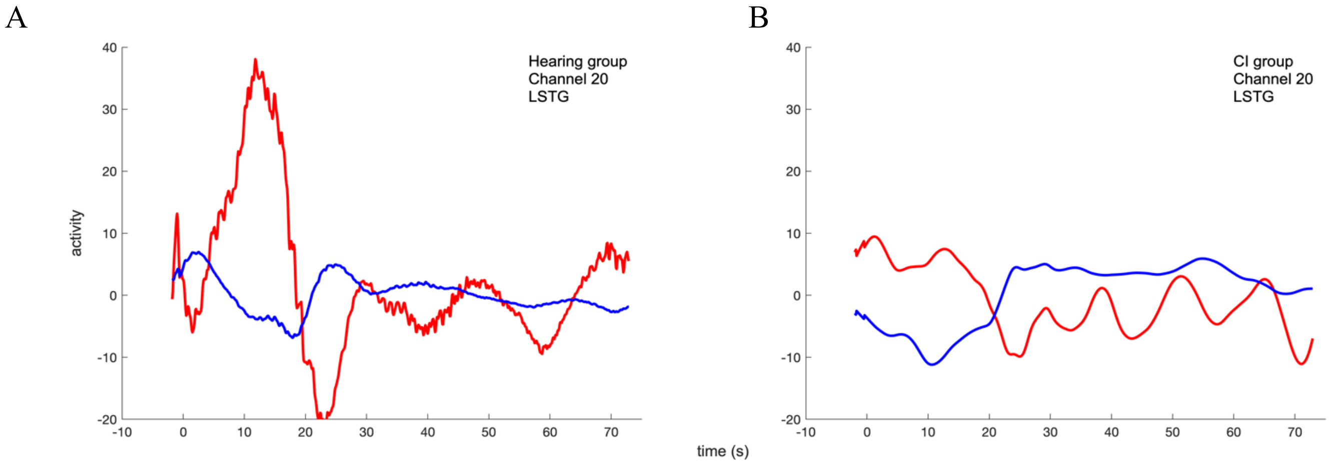

Note. The whole block interval (60s task+15s rest=75s) was plotted to demonstrate the response returns to baseline. The strength of the group-level hemodynamic response in channel 20 is comparatively lower in CI users than in hearing participants, indicating the influence of individual differences such as the age of language exposure through CI/ASL. It is important to note that while these HRFs provide insights into group-level responses, differences between the CI and hearing groups should be interpreted with caution due to variations in fNIRS data acquisition systems between the two groups.
